# Genomic Analyses Identify Manganese Homeostasis as a Driver of Group B Streptococcal Vaginal Colonization

**DOI:** 10.1128/mbio.00985-22

**Published:** 2022-06-06

**Authors:** Lindsey R. Burcham, Madeline S. Akbari, Norhan Alhajjar, Rebecca A. Keogh, Jana N. Radin, Thomas E. Kehl-Fie, Ashton T. Belew, Najib M. El-Sayed, Kevin S. McIver, Kelly S. Doran

**Affiliations:** a Department of Immunology and Microbiology, University of Colorado School of Medicine, Aurora, Colorado, USA; b Department of Microbiology, University of Illinois at Urbana-Champaign, Urbana, Illinois, USA; c Carl R. Woese Institute for Genomic Biology, University of Illinois at Urbana-Champaign, Urbana, Illinois, USA; d Cell Biology and Molecular Genetics, University of Maryland, College Park, Maryland, USA; e Center for Bioinformatics and Computational Biology, University of Maryland, College Park, Maryland, USA; University of Illinois at Chicago

**Keywords:** Group B Streptococcus, intrauterine infection, manganese, vaginal colonization

## Abstract

Group B Streptococcus (GBS) is associated with severe infections *in utero* and in newborn populations, including pneumonia, sepsis, and meningitis. GBS vaginal colonization of the pregnant mother is an important prerequisite for transmission to the newborn and the development of neonatal invasive disease; however, our understanding of the factors required for GBS persistence and ascension in the female reproductive tract (FRT) remains limited. Here, we utilized a GBS *mariner* transposon (*Krmit*) mutant library previously developed by our group and identified underrepresented mutations in 535 genes that contribute to survival within the vaginal lumen and colonization of vaginal, cervical, and uterine tissues. From these mutants, we identified 47 genes that were underrepresented in all samples collected, including *mtsA*, a component of the *mtsABC* locus, encoding a putative manganese (Mn^2+^)-dependent ATP-binding cassette transporter. RNA sequencing analysis of GBS recovered from the vaginal tract also revealed a robust increase of *mtsA* expression during vaginal colonization. We engineered an Δ*mtsA* mutant strain and found by using inductively coupled plasma mass spectrometry that it exhibited decreased concentrations of intracellular Mn^2+^, confirming its involvement in Mn^2+^ acquisition. The Δ*mtsA* mutant was significantly more susceptible to the metal chelator calprotectin and to oxidative stressors, including both H_2_O_2_ and paraquat, than wild-type (WT) GBS. We further observed that the Δ*mtsA* mutant strain exhibited a significant fitness defect in comparison to WT GBS *in vivo* by using a murine model of vaginal colonization. Taken together, these data suggest that Mn^2+^ homeostasis is an important process contributing to GBS survival in the FRT.

## INTRODUCTION

Infant morbidity and mortality resulting from adverse pregnancy outcomes remain a significant health burden, even in developed countries (1, 2), and one of the leading causes of adverse pregnancy outcomes is intrauterine infection (3). Streptococcus agalactiae, also known as group B Streptococcus (GBS), is a Gram-positive bacterium that asymptomatically colonizes the lower gastrointestinal tract and vaginal tract of 25 to 30% of healthy women. However, during pregnancy, GBS poses a risk to the mother and the developing fetus, as it has potential to cause severe infections (4). In pregnant women, the presence of GBS in the vaginal tract, particularly at high burdens, can result in ascending spread through the reproductive tract, causing intrauterine infection (5, 6). This has been shown to contribute to miscarriage, stillbirth, and preterm premature rupture of membranes (PPROM) (7–9), with an estimated 3.5 million preterm births due to GBS infection per year (7).

In addition to the impact of GBS vaginal colonization on maternal and fetal morbidity and mortality, GBS remains a primary threat to newborn babies. Approximately 50 to 70% of vaginally delivered infants of GBS-colonized mothers will become colonized, and 1 to 2% of colonized infants develop GBS invasive diseases, including pneumonia, sepsis, and meningitis (10, 11). These cases result from ascending infection of the bacterium through the placental membranes to initiate infection *in utero*, or alternatively, GBS may be acquired during passage through the birth canal by aspiration of infected vaginal fluids (9). During intrauterine GBS infection, localized inflammatory reactions allow bacteria to invade either the blood vessels of the fetus or the amniotic fluid, where the bacteria can gain entry to the fetus by aspiration or direct invasion (3, 12). In rare cases, GBS can lead to maternal sepsis (13, 14). To combat the risk of GBS infection in newborns, many countries have implemented universal or risk-based screening and the use of prophylactic antibiotics administered to colonized pregnant mothers at the time of delivery (15). But before late gestational GBS screening occurs, the pregnant mother and the fetus are susceptible to GBS colonization and ascension to the higher reproductive tissues and penetration of the placental membranes, through largely unknown mechanisms.

Transposon sequencing (Tn-seq) provides a high-throughput method to determine how individual genes contribute to bacterial fitness by combining untargeted mutagenesis with next-generation sequencing (16). Recent advances in the field of streptococcal pathogenesis have utilized transposon sequencing to determine gene fitness and essentiality in several pathogens both *in vitro* and *in vivo* (17–25). Previous work from our laboratory performed the first transposon sequencing experiment in the presence of calprotectin, a metal-chelating protein complex that is abundant in the neutrophilic cytosol, to identify some of the genes involved in the global response to metal availability *in vitro* (26). In this study, we sought to use our saturated transposon mutant library in a murine model of GBS vaginal colonization to identify the systems important for GBS fitness in the female reproductive tract (FRT). We identified 535 mutants that were underrepresented in comparison to the input library and important for colonization of the vaginal lumen or invasion into the higher reproductive tissues. Assignment of clusters of orthologous groups (COGs) revealed a majority of these underrepresented systems to contribute to nutrient transport or transcriptional regulation. We further identified that *mtsA*, the gene encoding a putative Mn^2+^-binding protein, was one of 47 genes that were underrepresented in all sites within the reproductive tract and contributes to Mn^2+^ import, survival during calprotectin-mediated metal chelation, resistance to oxidative stress, and GBS vaginal colonization. Collectively, these data support the conclusion that Mn^2+^ homeostasis is important for evasion of the host response and fitness in the FRT.

## RESULTS

### Genome-wide analysis of GBS factors involved in colonization and ascending infection.

**FIG 1:**
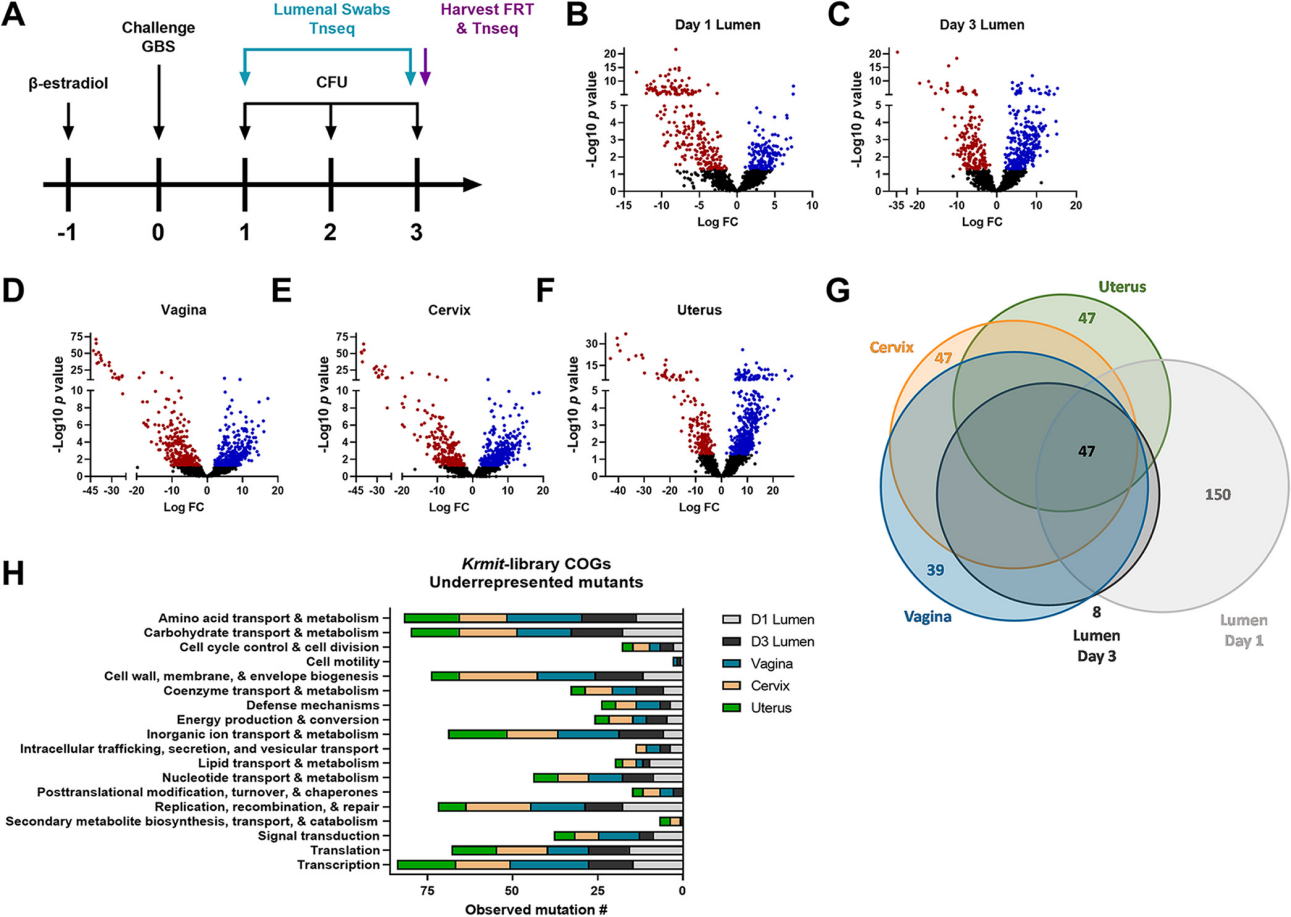
Determinants of GBS fitness in the female vaginal tract. (A) Model of transposon mutagenesis sample collection. (B to F) Volcano plots of underrepresented (red), overrepresented (blue), or unchanged (black) gene insertion mutations in the vaginal lumen at days 1 (B) and 3 (C) post-colonization or in vaginal (D), cervical (E), or uterine (F) tissues. (G and H) Venn diagram (G) and clusters of orthologous groupings (COGs) (H) as determined by EggNOG 5.0 for underrepresented mutations from all sample locations of underrepresented mutations.

To determine the systems important for GBS fitness in the FRT, we utilized our previously established murine model of vaginal colonization (4, 27). Triplicate groups of mice (*n *= 3/group) were synchronized with 17β-estradiol, and mice were challenged intravaginally with triplicate mid-logarithmic cultures of 10^7^ CFU of saturated *Krmit* transposon library. On subsequent days, vaginal swabs were collected, and on day 3 post-colonization, reproductive tissues were harvested. All samples were plated on selective medium to assess colonization and to recover transposon mutants for sequencing analysis (Fig. 1). Briefly, recovered transposon mutants from luminal swabs collected on days 1 and 3 and vaginal, cervical, and uterine tissues harvested day 3 post-colonization were pooled, and genomic DNA was isolated. Genomic DNA samples were sheared and enriched for *Krmit* transposon insertions by PCR, and Illumina adaptors were added (see Fig. S1A in the supplemental material). Samples were sequenced as previously described (26), reads were mapped to the Streptococcus agalactiae CJB111 genome (28), and differential analyses were performed using Tn-seq TRANSIT and DESeq2 by comparing *in vivo* samples to the input library (26, 29).

10.1128/mbio.00985-22.1 FIG S1 *In vivo Krmit*-library screen. (A) Schematic of transposon sequencing mutagenesis screen. (B and C) Venn diagram (B) and COGs (C) of overrepresented mutations from all sample locations. Download FIG S1, TIF file, 1.0 MB. Copyright © 2022 Burcham et al. 2022 Burcham et al. https://creativecommons.org/licenses/by/4.0/ This content is distributed under the terms of the Creative Commons Attribution 4.0 International license.

We detected a total of 535 underrepresented mutants with a significant defect in colonization of the vaginal lumen and/or higher reproductive tissues (Fig. 1B to G; Table S1). We identified underrepresented mutations in 250 genes in luminal samples collected day 1 post-colonization (Fig. 1B) and 192 genes in day 3 post-colonization samples (Fig. 1C). We further identified mutations in the samples collected from the reproductive tissues, with underrepresented mutations occurring in 277 genes in vaginal tissues, 237 genes in cervical tissues, and 182 genes in uterine tissues (Fig. 1D to F). Of the Tn mutants that were identified as underrepresented in the FRT, clusters of orthologous groupings (COGs) were identified for 145 and 117 genes in the luminal samples from days 1 and 3 post-colonization, respectively, and for 158, 149, and 101 genes in the vaginal, cervical, and uterine tissues, respectively (Fig. 1H; Fig. S2). The most highly represented COGs of known function for the luminal samples were those pertaining to carbohydrate and amino acid transport and metabolism and to replication, recombination, and repair, while the vaginal, cervical, and uterine tissues had the highest abundance for COGs involved in transcription, cell wall biogenesis, and inorganic ion transport and metabolism (Fig. 1H). We also observed overrepresented mutations, including 53 genes that were overrepresented for transposon insertion mutations in all samples collected (Fig. S1B), with COGs showing contribution of these genes to nutrient transport, transcription, and translation (Fig. S1C).

10.1128/mbio.00985-22.2 FIG S2 COG distribution by *in vivo* samples. COGs were assigned using eggNOG 5.0 for underrepresented mutants from each group of samples, including vaginal lumen on day 1 (A) and day 3 (B) post-colonization and reproductive tissues from the vagina (C), cervix (D), and uterus (E) on day 3 pos-tcolonization. Download FIG S2, TIF file, 1.0 MB. Copyright © 2022 Burcham et al. 2022 Burcham et al. https://creativecommons.org/licenses/by/4.0/ This content is distributed under the terms of the Creative Commons Attribution 4.0 International license.

10.1128/mbio.00985-22.6 TABLE S1 Fitness data from GBS transposon mutagenesis sequencing during murine vaginal colonization and ascending infection. Download Table S1, XLSX file, 0.1 MB. Copyright © 2022 Burcham et al. 2022 Burcham et al. https://creativecommons.org/licenses/by/4.0/ This content is distributed under the terms of the Creative Commons Attribution 4.0 International license.

**FIG 2:**
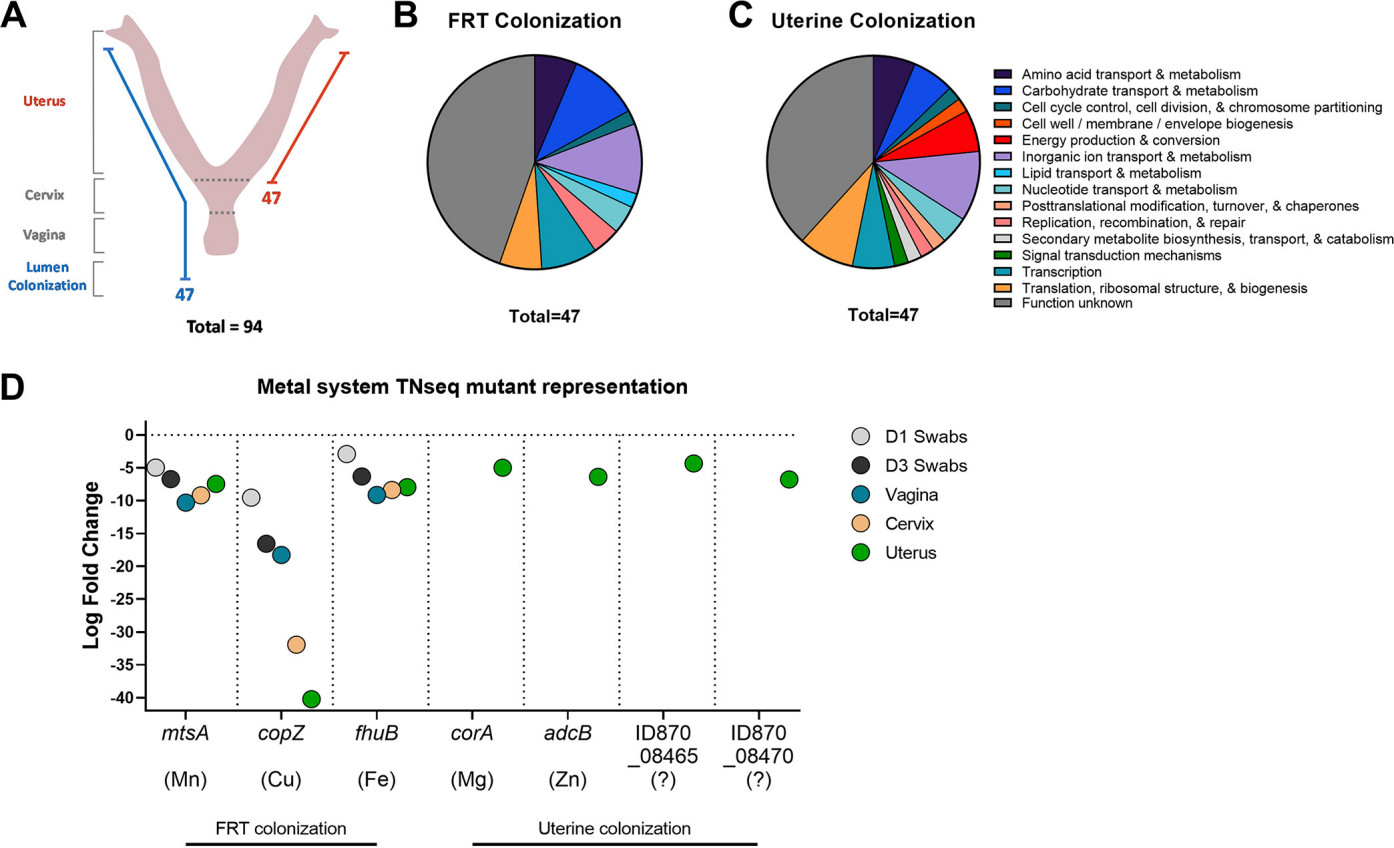
Important GBS factors for colonization of the vaginal lumen or uterus (A to C). *In vivo* Tn-seq identified (A) 94 unique genes and corresponding COGs that are important for colonization of the (B) FRT or (C) uterus. (D) Several systems involved in metal transport mutations were underrepresented and important for colonization of the FRT and uterus.

From these analyses, we observed underrepresented mutations in several systems that are involved or hypothesized to contribute to GBS colonization or virulence potential (Table 1), including pili, the newly characterized type VII secretion system (30), hydrolases, proteases, and bacteriocin immunity proteins. We also identified a large number of underrepresented mutations in systems involved in sensing and responding to environmental cues, including transcriptional regulators, two-component systems, phosphotransferase (PTS) systems, ATP-binding cassette transporters (ABC transporters), and metal homeostasis systems (Table 1). To understand the factors required specifically for GBS colonization of the vaginal lumen or the uterus, we further categorized the genes by those that were significantly underrepresented in the vaginal lumen and in all subsequent tissues (Fig. 2A, shown in blue) and those that were uniquely underrepresented in the uterine tissues (Fig. 2A, shown in red). We identified a unique set of 47 genes with underrepresented mutations detected in all samples collected (Table S2), indicating that GBS luminal persistence is an important prerequisite for invasion into the reproductive tissues. An additional 47 genes were uniquely found to contribute to penetrating the cervicovaginal barrier and accessing the uterus (Table S3). Similar COG classifications were found to be important for colonization of both the vaginal lumen and the uterus, including amino acid and carbohydrate metabolism and inorganic ion transport and metabolism (Fig. 2B and C). We identified unique COGs for each set of samples, with lipid transport and metabolism unique to colonization of all the FRT (Fig. 2B) and cell wall biogenesis, energy production and conversion, and signal transduction mechanisms unique to uterine colonization (Fig. 2C). From these subsets of data, we identified systems involved in the maintenance of copper, iron, Mn^2+^, zinc, and magnesium homeostasis as differentially important for colonization of specific sites in the FRT (Fig. 2D). One of the genes that was significantly underrepresented in the lumen and all tissues was *mtsA* (Fig. 2D). *mtsA* encodes the substrate-binding protein of a putative Mn^2+^/Fe-dependent ATP-binding cassette transporter, MtsABC. A previous study performed RNA sequencing of GBS strain A909 recovered from murine vaginal lavage fluid on day 2 post-colonization and found that *mtsA* was upregulated in the vaginal lumen (31). We performed a similar experiment using the GBS CJB111 strain and sequenced bacteria recovered from vaginal swab samples collected days 1 and 3 post-colonization and also observed a robust induction of *mtsA* at both time points (Fig. S3; Table S4). We provide the complete data set of our RNA sequencing experiment with CJB111 as an additional community resource (Table S4).

**TABLE 1:** Underrepresented mutants of interest identified by *in vivo* GBS transposon mutagenesis sequencing during murine vaginal colonization and ascending infection^*a*^

Functional category and gene no. (CJB111 genome)	Gene name	Description	Fold change in reproductive tract				
			D1	D3	VG	CX	UT
Transcriptional regulators							
ID870_00525		CtsR family transcriptional regulator				−4.93	
ID870_00635		LacI family DNA-binding transcriptional regulator		−9.94	−11.33		−25.05
ID870_00850		MerR family transcriptional regulator	−5.10				
ID870_00980		Transcriptional regulator	−8.19				
ID870_01280		LacI family DNA-binding transcriptional regulator			−5.36		
ID870_02045		LysR family transcriptional regulator		−5.52			
ID870_02800		LysR family transcriptional regulator	−9.51	−7.83	−8.05		
ID870_02815	*pyrR*	Transcriptional regulator		−4.35			
ID870_03090		TetR/AcrR family transcriptional regulator	−8.59				
ID870_03750		Helix-turn-helix transcriptional regulator	−6.92	−6.59	−9.99		
ID870_04400		Spx/MgsR family RNA polymerase-binding regulatory protein		−6.29	−6.05	−4.13	−5.68
ID870_05775		FadR family transcriptional regulator	−6.22	−8.42	−10.78	−9.33	−13.07
ID870_06770		ArgR family transcriptional regulator	−8.12	−12.14	−18.06	−17.19	−11.94
ID870_07170		AraC family transcriptional regulator		−4.38	−9.28	−7.60	−9.65
ID870_07370	*perR*	Peroxide-responsive transcriptional repressor			−5.04		
ID870_07715		Sugar-binding transcriptional regulator			−3.28	−2.35	
ID870_08260		Helix-turn-helix transcriptional regulator	−4.81				
ID870_08350		Helix-turn-helix transcriptional regulator	−8.81				
ID870_09330		MurR/RpiR family transcriptional regulator					−8.69
ID870_10200	*argR*	Arginine repressor					−7.70
ID870_10525		PadR family transcriptional regulator			−3.13	−6.26	−5.00
							
Two-component systems							
ID870_00210	*fspR*	Response regulator (TCS-16)			−4.78	−4.48	−7.15
ID870_00330		Response regulator (TCS-15)			−6.19		
ID870_03010		HAMP domain-containing histidine kinase (TCS-12)	−2.79				
ID870_04300	*ltdR*	LytTR family transcriptional regulator			−5.35		−7.56
ID870_05675	*yycF*	Response regulator transcription factor (TCS-8)	−3.92				
ID870_07355	*saeS*	HAMP domain-containing histidine kinase	−2.66		−6.47	−7.61	−4.19
ID870_07360	*saeR*	Response regulator transcription factor			−5.89		
ID870_08500	*lytS*	Sensor protein			−3.68		
ID870_10420		HAMP domain-containing histidine kinase (TCS-19)					−5.24
							
Metal systems							
ID870_00250	*adcAII*	Zinc ABC transporter substrate-binding protein				−5.66	
ID870_02005	*mtsR*	Metal-dependent transcriptional regulator	−4.89				
ID870_02010	*mtsA*	Metal ABC transporter substrate-binding protein	−4.95	−6.71	−10.29	−9.19	−7.44
ID870_02080	*nikC*	ABC transporter permease		−7.11	−10.16	−7.95	
ID870_02090	*nikE*	ABC transporter ATP-binding protein		−6.23	−7.75	−5.92	−7.57
ID870_02665	*fhuB*	Iron ABC transporter permease	−2.89	−6.31	−9.13	−8.38	−7.94
ID870_07025		Magnesium transporter CorA family protein					−4.98
ID870_07140	*sczA*	MerR family transcriptional regulator			−11.26	−23.68	
ID870_07395	*copZ*	Heavy-metal-associated domain-containing protein	−9.54	−16.55	−18.27	−31.92	−40.23
ID870_08465		ABC transporter ATP-binding protein					−4.34
ID870_08470		ABC transporter permease					−6.77
ID870_08640	*adcB*	Zinc ABC transporter permease					−6.37
ID870_08780		Putative metal homeostasis protein	−10.07				
							
Transport systems							
Phosphotransferase systems							
ID870_00140		PTS transporter subunit EIIC					−4.92
ID870_00190		PTS sugar transporter subunit IIB	−9.85				
ID870_00265		PTS sugar transporter subunit IIA		−6.35	−7.73	−8.63	−5.88
ID870_00430		PTS system mannose/fructose/*N*-acetylgalactosamine-transporter subunit IIB	−7.30				
ID870_00990		PTS fructose transporter subunit IIB			−9.43	−19.55	−21.30
ID870_00995		PTS sugar transporter subunit IIA	−7.71		−6.94	−6.60	
ID870_01290		PTS glucose transporter subunit IIA		−3.29	−3.53	−3.21	
ID870_01665		Transporter substrate-binding domain-containing protein		−6.37	−6.37	−5.75	−4.38
ID870_07535		PTS mannose/fructose/sorbose transporter family subunit IID	−3.95	−6.86	−8.25	−10.86	−30.53
							
ATP-binding cassette transporters							
ID870_01555		ECF-type riboflavin transporter substrate-binding protein					−3.90
ID870_01670		Amino acid ABC transporter permease			−8.20	−9.11	−9.11
ID870_01820		ABC transporter ATP-binding protein		−2.92	−3.35		
ID870_02315		Amino acid ABC transporter ATP-binding protein			−5.60		−7.11
ID870_02480		Transporter substrate-binding domain-containing protein	−3.74	−7.05	−10.51	−11.00	−12.24
ID870_02675		ABC transporter ATP-binding protein	−2.53	−5.25	−8.06	−5.70	−4.94
ID870_02990		ABC transporter ATP-binding protein		−3.51	−5.86	−3.42	
ID870_04360		ABC transporter ATP-binding protein			−4.48		
ID870_04650		ABC transporter permease		−5.64	−7.43	−4.99	
ID870_04655		ABC transporter ATP-binding protein			−4.63		
ID870_05620		ABC transporter ATP-binding protein		−5.38	−6.01	−8.57	
ID870_05695		Amino acid ABC transporter permease	−3.81				
ID870_05935		ABC transporter ATP-binding protein	−7.10				
ID870_05965		ABC transporter permease	−2.69		−10.28	−9.16	−7.53
ID870_06520		ABC transporter permease			−3.53		
ID870_06635		ECF transporter S component		−2.76	−3.63		
ID870_07335	*bioY*	Biotin transporter					−8.85
ID870_07465		ABC transporter ATP-binding protein				−4.25	
ID870_07805		ABC transporter permease			−10.09	−5.43	
ID870_07885		Amino acid ABC transporter ATP-binding protein	−5.20	−8.84	−12.51	−10.59	−11.97
ID870_09850		Energy-coupling factor transporter transmembrane protein EcfT	−3.91				
ID870_10515		ABC transporter permease			−3.21		
							
Virulence potential							
ID870_01235	*comE*	Competence protein			−8.79		−7.62
ID870_02600	*pilB*	PI-2a pilus major subunit		−3.57		−3.52	
ID870_02605		Class C sortase			−6.62	−4.78	
ID870_02615	*pilC*	PI-2a pilus subunit	−4.25		−8.97	−8.78	
ID870_03655		LrgB family protein		−8.42	−13.29	−10.63	−9.08
ID870_04185	*essA*	Type VII secretion protein	−3.25				
ID870_04625		Class A sortase		−4.69	−9.12	−8.51	−7.60
ID870_05305		Serine hydrolase		−7.42	−10.17	−9.95	−8.09
ID870_06020	*srtC1*	Class C sortase		−6.09	−9.97	−6.00	
ID870_07305		Serine protease			−10.70	−10.35	−9.43
ID870_07950		Bacteriocin immunity protein	−2.85				
ID870_08585		Type II secretory pathway, pseudopilin PulG	−11.66	−7.57	−9.51		
ID870_09920	*ebhA*	EbhA				−4.58	
ID870_09980		Virulence-associated protein E				−3.88	
ID870_10015		Type II toxin-antitoxin system RelB/DinJ family antitoxin	−8.12				
ID870_10190		Bacteriocin immunity protein	−2.78				−6.92
ID870_10550		Type II toxin-antitoxin system RelB/DinJ family antitoxin	−8.65				
ID870_10565		WXG100 family type VII secretion target			−4.75	−4.71	−6.38

a D1, vaginal lumen on day 1 postcolonization; D3, vaginal lumen on day 3 postcolonization; VG, tissue from the vagina; CX, tissue from the cervix; UT, tissue from the uterus.

10.1128/mbio.00985-22.3 FIG S3 *In vivo* RNA sequencing from GBS days 1 and 3 post-colonization. Volcano plots of downregulated (red), upregulated (blue), and unchanged (gray) genes from GBS recovered from the vaginal lumen on day 1 (A) and day 3 (B) post-colonization. Significance was determined by differential expression analyses comparing *in vivo* samples to culture input samples, with statistical cutoffs of ±2 log_2_ fold change and a *P *of <0.05. Download FIG S3, TIF file, 0.5 MB. Copyright © 2022 Burcham et al. 2022 Burcham et al. https://creativecommons.org/licenses/by/4.0/ This content is distributed under the terms of the Creative Commons Attribution 4.0 International license.

10.1128/mbio.00985-22.7 TABLE S2 Genes with underrepresented mutations important for colonization of the vaginal lumen. Download Table S2, DOCX file, 0.03 MB. Copyright © 2022 Burcham et al. 2022 Burcham et al. https://creativecommons.org/licenses/by/4.0/ This content is distributed under the terms of the Creative Commons Attribution 4.0 International license.

10.1128/mbio.00985-22.8 TABLE S3 Genes with underrepresented mutations important for uterine colonization. Download Table S3, DOCX file, 0.02 MB. Copyright © 2022 Burcham et al. 2022 Burcham et al. https://creativecommons.org/licenses/by/4.0/ This content is distributed under the terms of the Creative Commons Attribution 4.0 International license.

10.1128/mbio.00985-22.9 TABLE S4 RNA sequencing data from GBS CJB111 during murine vaginal colonization. Download Table S4, XLSX file, 0.1 MB. Copyright © 2022 Burcham et al. 2022 Burcham et al. https://creativecommons.org/licenses/by/4.0/ This content is distributed under the terms of the Creative Commons Attribution 4.0 International license.

### Role of *mtsA* in Mn^2+^ homeostasis.

**FIG 3:**
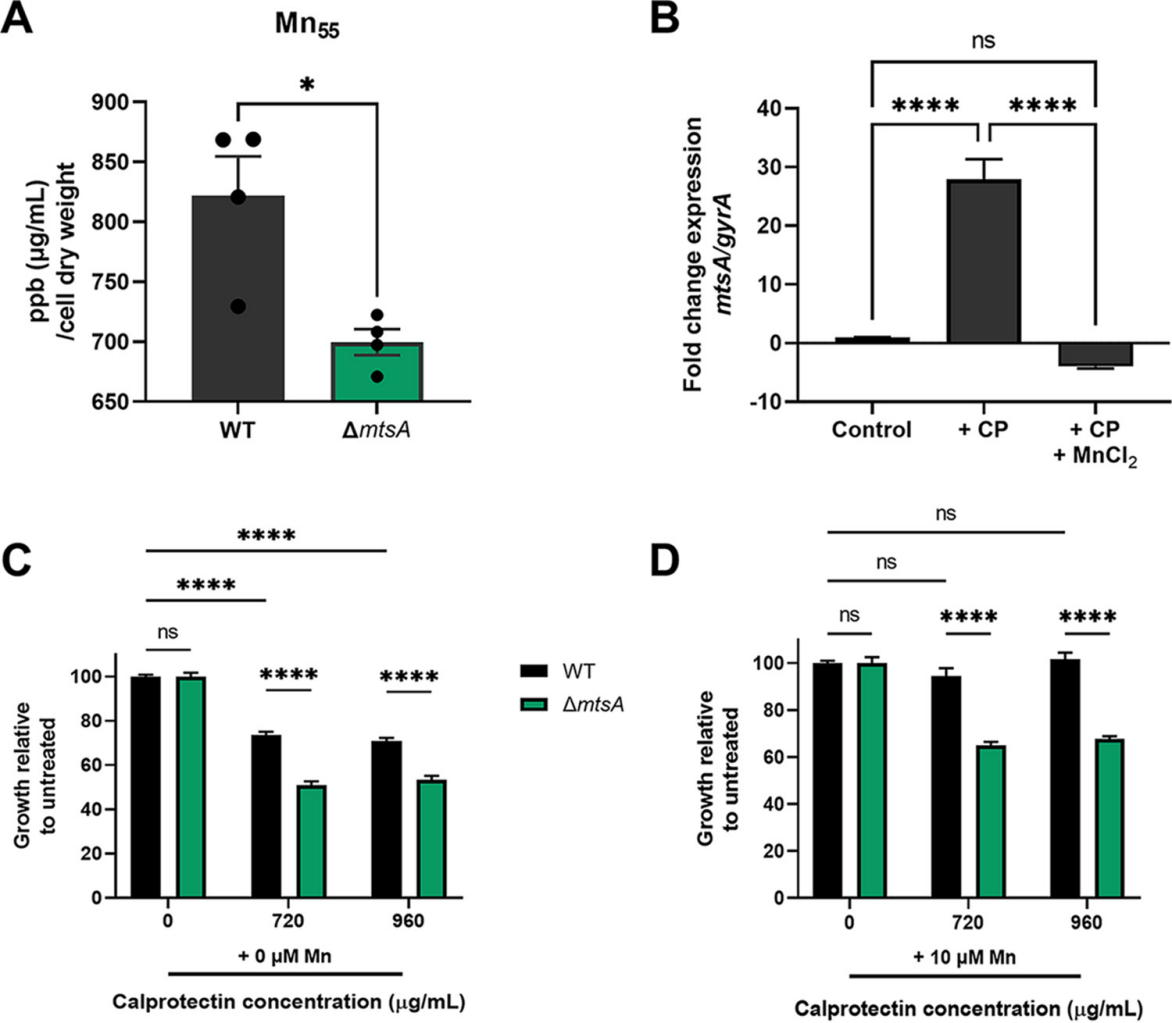
GBS *mtsA* contributes to Mn^2+^ import and survival during calprotectin stress *in vitro*. (A) Intracellular Mn_55_ was determined for GBS CJB111 WT and Δ*mtsA* by ICP-MS. (B) Quantitative RT-PCR was used to assess expression of *mtsA* following exposure to 960 μg/mL calprotectin with or without supplementation with 50 μM MnCl_2_. Fold change was calculated by ΔΔ*C_T_* analysis, with *gyrA* serving as the internal control. Data are displayed as the average fold change in expression from three independent experiments. (C and D) Growth of GBS WT and Δ*mtsA* strains in the presence of 720 to 960 μg/mL calprotectin (C) or 720 to 960 μg/mL calprotectin with 10 μM MnCl_2_ supplementation (D). Significance was determined by an unpaired two-tailed Student's *t* test (A), one-way ANOVA with Tukey’s multiple-comparison test (B), or two-way ANOVA with Tukey’s multiple-comparison test (C and D). *, *P < *0.05; ****, *P < *0.0001; ns, not significant.

To confirm the importance of *mtsA* to Mn^2+^ homeostasis, we constructed a Δ*mtsA* mutant strain as described in Materials and Methods. We first performed inductively coupled plasma mass spectrometry (ICP-MS) on bacterial pellets grown in Todd-Hewitt broth (THB) medium as described in Materials and Methods. The Δ*mtsA* mutant had significantly lower intracellular concentrations of Mn^2+^ than the wild-type (WT) GBS control, with a mean of 821.9 μg/mL and a standard error of the mean (SEM) of ±32.8 μg/mL in the WT strain and a mean of 699.7 μg/mL and a SEM of ±10.88 μg/mL in the Δ*mtsA* mutant strain, with all strains normalized by cell dry weight (mg) (Fig. 3A). The levels of intracellular Mn^2+^ were restored in the complemented (Δ*mtsA*+) strain compared to the WT GBS control (Fig. S4A). These data support the hypothesis of the involvement of *mtsA* in Mn^2+^ transport. Previous work from our laboratory demonstrated that calprotectin, a protein complex produced by the host, inhibited GBS growth by limiting nutrient metal ions (26). Calprotectin is known to possess two metal-binding sites, and previous studies have described site 1 to form a unique His_6_ site that serves as a high-affinity binding site for Mn^2+^ ions (32–34). We next hypothesized that expression of genes involved in Mn^2+^ acquisition would increase following exposure to calprotectin. We therefore assessed expression of *mtsA* by quantitative reverse transcriptase PCR (qRT-PCR) analysis following treatment with calprotectin or with calprotectin plus MnCl_2_ supplementation. Fold changes in *mtsA* expression were calculated by the ΔΔ*C_T_* method with *gyrA* serving as an internal control and were compared to expression observed in untreated controls. Following exposure to calprotectin, we observed a significant induction of *mtsA* expression, nearly 28-fold upregulation, while supplementation with MnCl_2_ following exposure to calprotectin restored expression to control levels, at nearly 4-fold downregulation compared to that of untreated controls (Fig. 3B). Having confirmed the importance of *mtsA* to Mn^2+^ import, we then hypothesized that the Δ*mtsA* mutant strain would exhibit increased sensitivity to chelation by calprotectin. We assessed growth (as measured by optical density at 600 nm [OD_600_]) of GBS WT and Δ*mtsA* mutant strains in the presence of 720 and 960 μg/mL calprotectin with or without the addition of 10 μM MnCl_2_ (Fig. 3C and D). Similar to what was previously described by our laboratory (26), we observed a significant inhibition of growth in WT CJB111 grown in the presence of calprotectin (Fig. 3C), and further, we identified a significant increase in susceptibility to calprotectin-mediated metal chelation in the Δ*mtsA* mutant strain compared to that of WT GBS (Fig. 3C). Notably, we found that supplementing all samples with 10 μM MnCl_2_ rescued growth of WT GBS grown in the presence of calprotectin, while growth was still impaired in the GBS Δ*mtsA* mutant regardless of MnCl_2_ supplementation (Fig. 3D).

10.1128/mbio.00985-22.4 FIG S4 GBS WT and Δ*mtsA* dynamics *in vitro* and *in vivo.* (A) ICP-MS confirmation of intracellular Mn^55^ concentration in CJB111 WT, Δ*mtsA*, and Δ*mtsA*+ strains. (B) Cultures of WT and Δ*mtsA* strains were inoculated 1:1 in THB to assess competition *in vitro*. (C to F) Female CD-1 mice were colonized with 1 × 10^7^ CFU of GBS A909 WT or Δ*mtsA* in a single challenge. Recovered CFU were quantified from vaginal lavage fluid over time (C) or from vaginal (D), cervical (E), or uterine (F) tissue homogenates harvested on day 6 post-colonization. Significance was determined by two-way ANOVA with Sídák’s multiple-comparison test (A and B) or an unpaired two-tailed Student’s *t* test (C to E). **, *P < *0.01; ***, *P < *0.001; ****, *P < *0.0001; ns, not significant. Download FIG S4, TIF file, 0.6 MB. Copyright © 2022 Burcham et al. 2022 Burcham et al. https://creativecommons.org/licenses/by/4.0/ This content is distributed under the terms of the Creative Commons Attribution 4.0 International license.

### Mn^2+^ transport as an important driver of GBS vaginal colonization.

**FIG 4:**
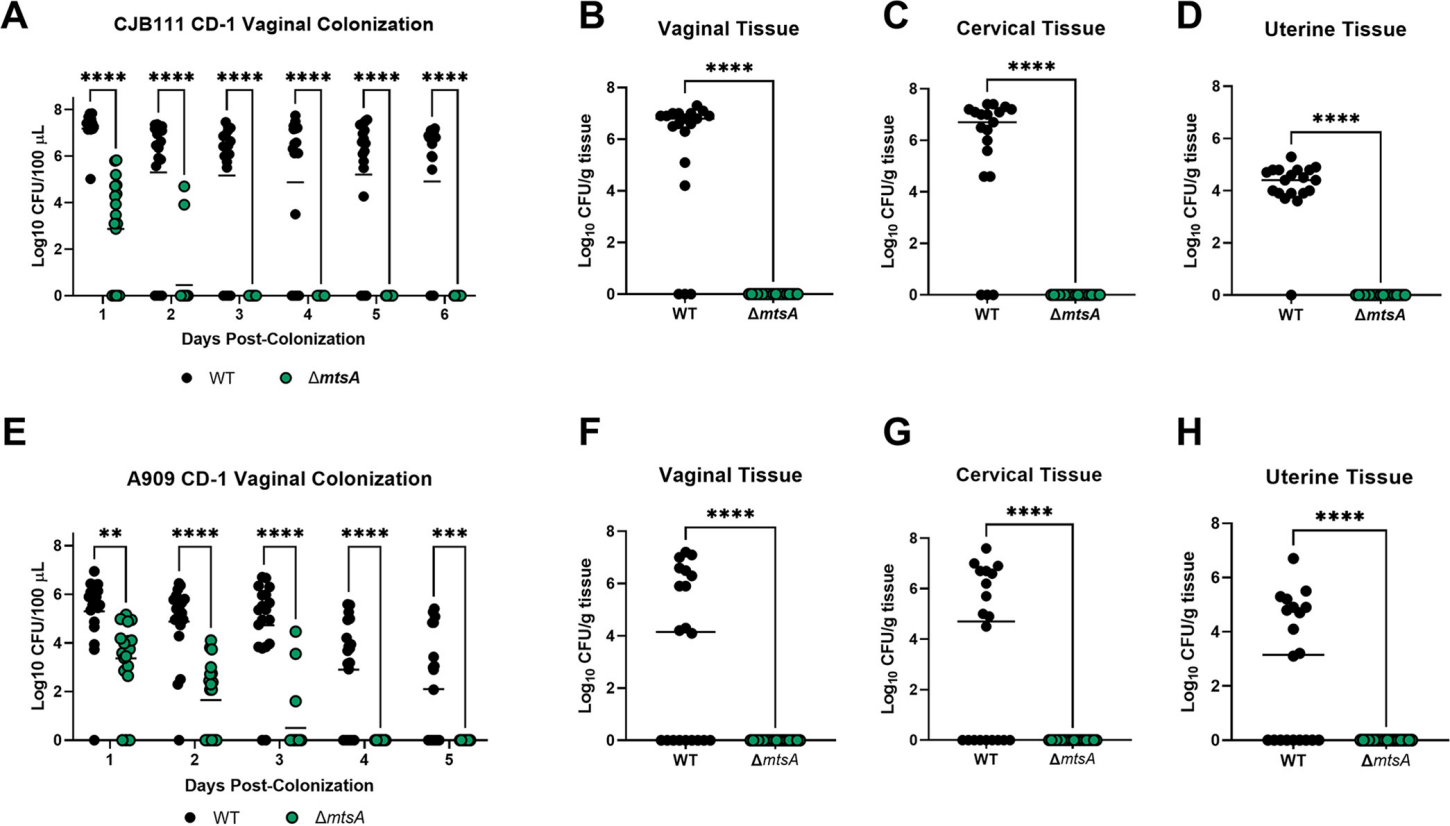
GBS Mn^2+^ homeostasis contributes to vaginal colonization *in vivo*. Female CD-1 mice were colonized with 1 × 10^7^ CFU of both GBS WT and Δ*mtsA* strains. WT and mutant strains were tested in both the CJB111, serotype V (A to D), and A909, serotype Ia (E to H), genetic lineages. Recovered CFU were quantified from vaginal lavage fluid over time (A and E) or from vaginal (B and F), cervical (C and G), or uterine (D and H) tissue homogenates harvested on day 6 post-colonization. Significance was determined by two-way ANOVA with Sídák’s multiple-comparison test (A and E) or an unpaired two-tailed Student's *t* test (B to D and F to H). **, *P < *0.01; ****, *P < *0.0001.

**FIG 5:**
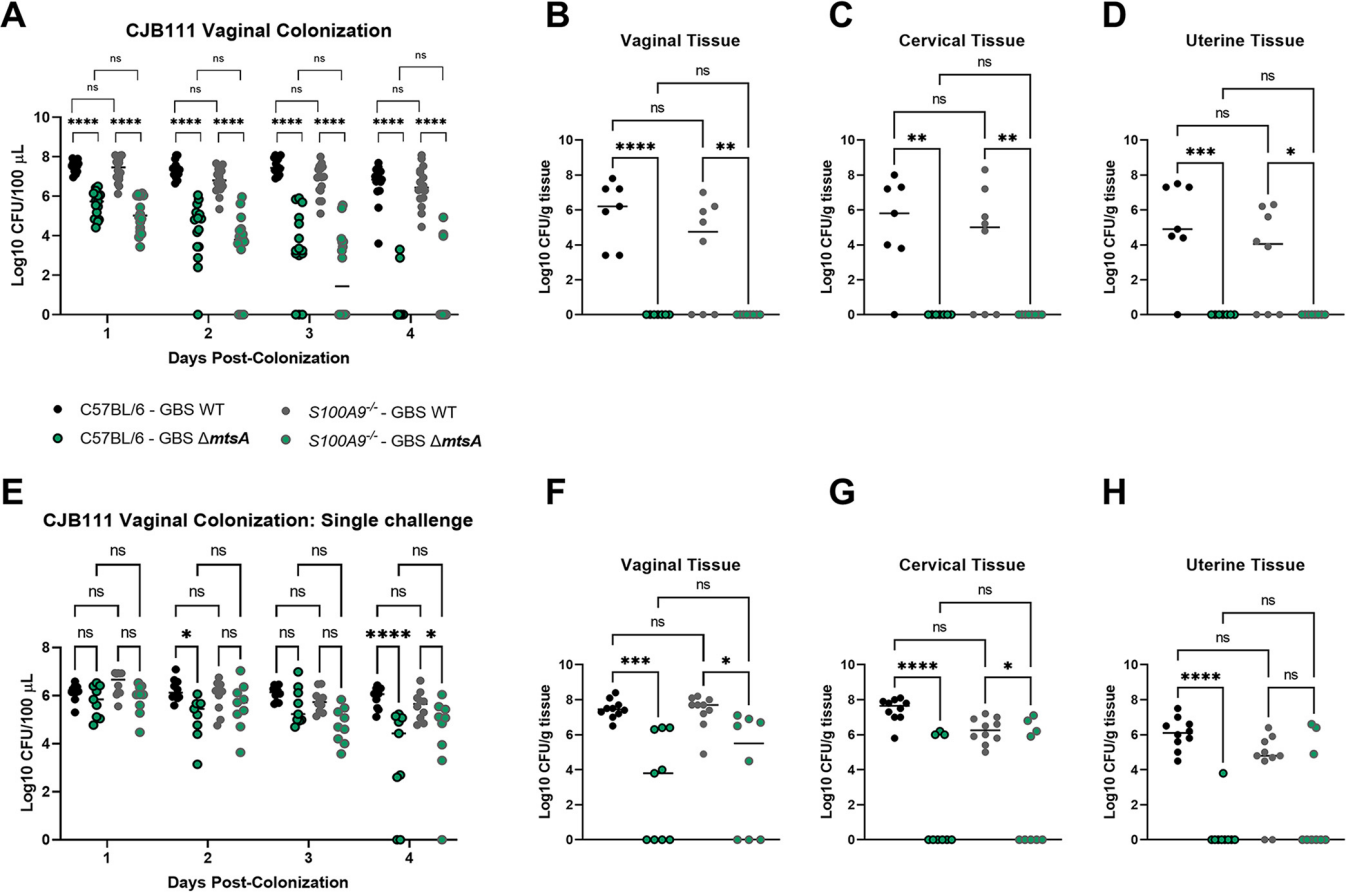
GBS Mn^2+^ homeostasis is independent of host calprotectin stress in the FRT. Female C57BL/6 and *S100A9^−/−^* mice were colonized with 1 × 10^7^ CFU of GBS CJB111 WT and Δ*mtsA* strains in competition (A to D) or as single challenge (E to H). Recovered CFU were quantified from vaginal lumen (A and E) or vaginal (B and F), cervical (C and G), or uterine (D and H) tissue homogenates. Significance was determined by two-way ANOVA with Sídák’s multiple-comparison test (A and E) or one-way ANOVA with Sídák’s multiple-comparison test (B to D and F to H). *, *P < *0.05; **, *P < *0.01; ***, *P < *0.001; ****, *P < *0.0001; ns, not significant.

The findings reported thus far led us to hypothesize that MtsA would contribute to GBS colonization and persistence within the FRT. Using our previously described murine model of vaginal colonization, we colonized female CD-1 mice intravaginally with both the WT CJB111 (serotype V) GBS and the Δ*mtsA* mutant strain in competition. The Δ*mtsA* mutant strain exhibited a significant fitness defect compared to WT GBS as early as day 1 post-colonization (Fig. 4A). By day 3 post-colonization, the WT strain had outcompeted the Δ*mtsA* mutant and all 19 mice had cleared the Δ*mtsA* mutant strain from the vaginal lumen, while 14/19 mice were still colonized with WT CJB111 at the experimental endpoint (Fig. 4A). To assess GBS burden in the higher reproductive tissues, we harvested tissues on day 6 post-colonization and enumerated GBS CFU. We observed a complete clearance of the Δ*mtsA* strain from the vagina, cervix, and uterus, although WT GBS was detected at a high burden in all tissues (Fig. 4B to D). We similarly found that the WT outcompeted the Δ*mtsA* mutant strain *in vitro* when grown in coculture (Fig. S4B). All vaginal colonization experiments were repeated in a second GBS genetic background, and we observed similar phenotypes in the serotype Ia WT A909 and A909 Δ*mtsA* strains (Fig. 4E to H). Consistent with what we observed in the CJB111 background, we detected complete clearance of the Δ*mtsA* mutant strain from the vaginal lumen at day 4 post-colonization, while WT A909 was recovered from the lumen of 8/19 mice at the time of tissue collection (Fig. 4E to H). We also observed during single challenge that the Δ*mtsA* mutant strain exhibited a reduced ability to colonize the FRT compared to the WT strain (Fig. S4C to F). To determine if phenotypes observed between the WT and the Δ*mtsA* mutant were due to the presence of calprotectin in the FRT during GBS colonization, we colonized age-matched WT C57BL/6 and *S100A9*^−/−^ calprotectin knockout mice with WT and Δ*mtsA* GBS in competition or in single challenge. We found that the Δ*mtsA* mutant strain still exhibited a significant decrease in fitness in colonization and ascension of both C57BL/6 WT and *S100A9*^−/−^ mice compared to the WT GBS during competition (Fig. 5A to D) or single challenge (Fig. 5E to H). These data confirm that GBS MtsA is important for colonization of the FRT, in both the presence and absence of calprotectin.

### Mn^2+^ homeostasis impacts GBS sensitivity to oxidative stressors.

**FIG 6:**
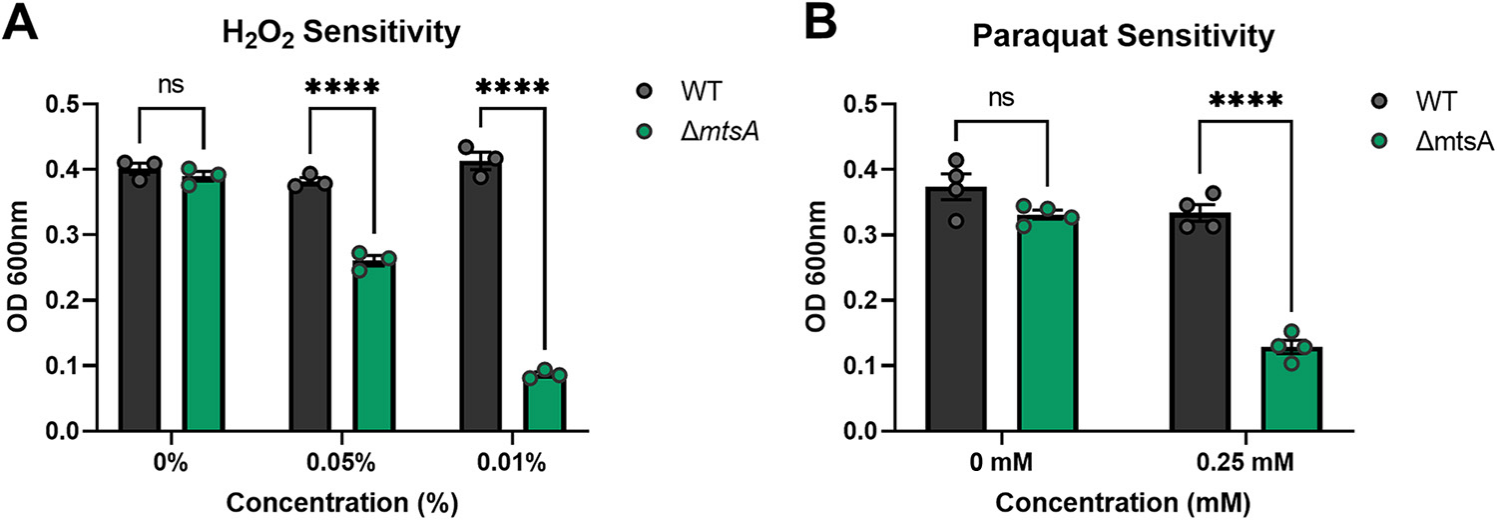
Role of Mn^2+^ homeostasis in GBS oxidative stress resistance. Growth of GBS WT and Δ*mtsA* strains in the presence of increasing concentrations of H_2_O_2_ (A) and paraquat (B). Significance was determined by two-way ANOVA with Sídák’s multiple-comparison test. ****, *P < *0.0001.

As Mn^2+^ homeostasis has been previously shown to contribute to the ability of bacterial pathogens to withstand oxidative stress (33, 35, 36), we hypothesized that the Δ*mtsA* mutant would exhibit increased sensitivity to stress. To understand the contribution of *mtsA* to GBS survival during oxidative stress, we assessed the growth of the WT and the Δ*mtsA* mutant strain following addition of H_2_O_2_ and the superoxide stressor paraquat. We observed a significant decrease in growth of the Δ*mtsA* mutant strain in the presence of both H_2_O_2_ (Fig. 6A) and paraquat (Fig. 6B) in comparison to that of WT GBS.

## DISCUSSION

Vaginal colonization by GBS is an important prerequisite for maternal and fetal infections. Despite the abundance of data showing the contribution of GBS to maternal-fetal morbidity and mortality, our understanding of the factors involved in colonization or ascension in the FRT remain to be fully elucidated. In this study, we sought to utilize a high-throughput transposon mutagenesis screen to identify systems involved in colonization of the lumen and invasion into the reproductive tissues using our previously described *mariner*-GBS transposon mutant library (26). From this *in vivo* screen, we identified 525 underrepresented mutants in systems involved in transcriptional regulation, cellular metabolism, and nutrient import and efflux and systems involved in GBS virulence. As *mtsA* transposon mutants were found to be underrepresented in all samples collected, we further characterized the role of *mtsA* in GBS survival during calprotectin and oxidative stress and persistence within the vaginal tract. These data provide new insight into the importance of metal homeostasis for bacterial colonization and ascension of the FRT.

GBS systems of particular interest that were identified as loss-of-function mutations, or underrepresented mutations, included regulatory systems such as two-component regulatory systems or transcriptional regulators (Table 1). We observed underrepresented mutations in several two-component systems (TCS) that were previously annotated, including TCS-8, -12, -15, -16, and -18 (37). Of these, few have been characterized in GBS and their regulon is unknown, with the exception of TCS-16 (FspRS). FspRS has been shown to be induced by and necessary for GBS growth in the presence of fructose-6-phosphate and is important for persistence within the murine vaginal tract (37). We also observed underrepresented mutations in SaeRS and LtdR, two recently studied TCS in GBS (31, 38). Expression of SaeRS was previously shown to be induced during GBS vaginal colonization (31), and mutations in genes regulated downstream of SaeRS led to significant decreases in GBS fitness in the vaginal tract (31). Further, LtdR is a response regulator that our laboratory has previously shown to contribute to regulation of virulence (38). Previous studies demonstrated that a Δ*ltdR* strain was cleared more quickly from the murine vaginal tract than WT GBS (38), confirming the underrepresentation we observed in our transposon screen.

In addition to systems involved in gene regulation, we also observed a significant underrepresentation of known and putative systems involved in GBS virulence. We identified underrepresented mutants in the lumen and in vaginal and cervical tissues in pilus components of the pilus island-2a (PI-2a), including the major and minor pilus subunits and the pilus 2a cell wall anchoring C class sortase. GBS strains encode three different pilus islands, PI-1, PI-2a, and PI-2b, with most strains expressing two different types. Studies from our laboratory have previously described the importance of GBS pili in interactions with reproductive epithelial cells and in vaginal colonization *in vivo* (39, 80). Interestingly, PI-1 was unaltered in our transposon mutagenesis screen, and it has been previously shown that PI-1 does not promote GBS interactions with the reproductive epithelium (40). The type VII secretion system was recently described in GBS and was shown to contribute to invasive disease through EsxA pore formation-induced endothelial cell cytotoxicity (30). Although the GBS type VII secretion system has not yet been shown to contribute to colonization of FRT, we observed underrepresented mutations in *essA*, a gene encoding a core machinery component, and in a gene encoding a putative effector WXG100 family protein in samples collected from the lumen and other tissues, likely suggesting an important role in the reproductive tract as well.

In addition to underrepresented mutations, we also detected mutations that are enriched or overrepresented during GBS colonization (see Table S1 in the supplemental material). Transposon sequencing screens provide a high-throughput approach to determine biological fitness in a given environment (16), although this competitive environment does not always correlate with data observed in single mutations. For example, our screen identified *hylB* as a gene with significantly overrepresented mutations (Table S1), suggesting that mutations in *hylB* increase GBS fitness in the female reproductive tract; however, it has been previously shown that hyaluronidase (encoded by *hylB*) is a secreted factor that contributes to vaginal colonization and reproductive tract ascension (41, 42). Another example of an overrepresented mutation found in our data set was *cylE*, the gene that encodes the GBS β-hemolysin/cytolysin. Interestingly, *cylE* mutants have previously been shown to exhibit a decreased persistence in a murine model of vaginal colonization (43). Thus, there are limitations to the sensitivity of transposon sequencing, specifically as it is unable to detect the contribution of individual mutations in genes encoding products secreted into the environment (44, 45). Also in this study, rather than declaring gene essentiality, we aimed to focus our analyses on GBS transposon mutant fitness. This could allow for some mutants that are classified herein as underrepresented to have essential roles *in vivo*, with some examples including DNA polymerase, tRNAs, and genes encoding ribosomal proteins.

Another group of genes that were well represented in our data set were systems involved in the maintenance of metal ion homeostasis. Some of the underrepresented metal-dependent systems of interest were those involved in homeostasis of zinc, copper, a putative nickel system, and Mn^2+^. Underrepresented mutations in the zinc transport machinery, including *adcAII* and *adcB* (26, 46, 47), were detected only in the cervical and uterine tissue samples, suggesting that the vaginal lumen may in fact be a zinc-replete environment where GBS may need not rely on these systems for fitness, although it is important to note that we and others identified genes involved in zinc import to be upregulated in the vaginal lumen (31) (Table S4). While previous work from our laboratory has shown that the zinc import machinery is crucial for the development of invasive disease *in vivo* (26), the contribution of these systems to GBS vaginal colonization has not yet been described. We also identified *copZ* as underrepresented in all samples collected. *CopZ* is a gene that encodes a copper-binding chaperone protein contributing to regulation of the *copY* repressor, and the downstream, *copA* are collectively involved in copper efflux (48–50). CopZ and the ortholog CupA of Streptococcus pneumoniae have been shown to reduce Cu^2+^ to Cu^1+^, which contributes to regulatory signaling, to growth in the presence of copper, and to interactions with host cells (51, 52). Components of the putative *nik* operon (ID870_02070 to ID870_02090), including *nikC* and *nikE*, were also underrepresented in luminal swabs, as well as vaginal, cervical, and uterine tissues, and were upregulated during vaginal colonization in the GBS CJB111 genetic background reported here and in a previously published data set in the GBS A909 background (31). The function of this putative system remains to be studied.

To maintain intracellular Mn^2+^ homeostasis, bacteria rely largely on ABC transport systems or natural resistance-associated macrophage proteins (NRAMPs) (35, 53–55). GBS expresses both the *mtsABC* and *mntH* systems. The GBS MtsABC transporter is conserved across GBS strains and other streptococcal pathogens (56); however, other pathogenic streptococci, including S. pyogenes and S. pneumoniae, are devoid of NRAMP transporters (57, 58). We detected an underrepresentation of mutations in *mtsA* across the lumen and all tissue samples collected, and previous analysis of the GBS transcriptome identified *mtsA* as strongly induced during vaginal colonization (31). We were able to confirm this robust induction of *mtsA* in another GBS background by performing RNA sequencing during vaginal colonization.

The host has also evolved an elegant strategy, termed nutritional immunity, to limit bioavailable metals from invading pathogens (59, 60). Calprotectin is formed by a heterodimer of two S100 family proteins, S100A8 and S100A9, and is a key mediator of this process. Calprotectin is a unique chelating molecule due to the presence of a distinctive His_6_ site that creates a high-affinity Mn^2+^ binding site (32, 33). Our laboratory has previously conducted a transposon mutant screen of GBS grown in the presence of calprotectin, and interestingly, several of the systems we observed as important *in vivo* were also deemed important for calprotectin survival *in vitro*, including the *mtsABC* system. We show here that the Δ*mtsA* mutant strain is more susceptible to chelation by calprotectin than WT GBS, and while supplementation of extracellular Mn^2+^ rescues growth of calprotectin-treated WT GBS, the Δ*mtsA* mutant strain remains susceptible to chelation despite Mn^2+^ supplementation. While our work has focused on the role of MtsA in Mn^2+^ homeostasis, in light of previous work showing the ability of calprotectin to also induce iron stress (61–63) and knowledge that expression of the GBS *mtsABC* locus is induced under both Mn^2+^ and iron limitation (64), it will be of interest to examine whether the *mtsABC* locus is also involved in iron uptake and how this might contribute to GBS colonization of the FRT.

The impact of bacterial Mn^2+^ homeostasis in the FRT had not been described. Here, we utilized a murine model of vaginal colonization previously established in our laboratory (4, 27) to compare fitness of WT GBS and the Δ*mtsA* mutant strains. Using two different GBS genetic backgrounds, CJB111 (serotype V) and A909 (serotype Ia), we demonstrate that the Δ*mtsA* mutant strain has a significant decrease in fitness in the FRT compared to WT GBS during competition and single challenge, confirming the importance of Mn^2+^ homeostasis for GBS vaginal colonization and persistence. Interestingly, we observed more dramatic phenotypes of the Δ*mtsA* mutant during competition with the WT strain both *in vitro* and *in vivo*. This suggests that manganese homeostasis is an important factor for GBS fitness and may also significantly contribute to GBS competition with other members of the vaginal microbiota. We further performed these animal studies in WT and *S100A9^−/−^* mice that lack functional calprotectin. We observed a similar decrease in fitness of the Δ*mtsA* mutant strain in *S100A9^−/−^* mice, suggesting that the availability of this metal remains limited even in the absence of calprotectin. These data could provide insight into the limitation of bioavailable Mn^2+^ within the vaginal mucosa, and studies on how the vaginal microbiota and host mucosal immunity impact Mn^2+^ levels warrants further investigation. Mn^2+^ is also a known metal ion cofactor for bacterial proteins that function in response to oxidative stress (35, 36, 65). This is consistent with our observation that the Δ*mtsA* mutant was significantly more sensitive to superoxide (paraquat) and H_2_O_2_ treatment. GBS is known to express a single Mn^2+^-dependent superoxide dismutase (66), and future studies will seek to elucidate the role of this and other GBS Mn^2+^-dependent enzymes during colonization of the FRT.

In this study, we utilized for the first time a saturated GBS transposon library during *in vivo* colonization to begin to understand the important factors for GBS persistence in the vaginal lumen and ascension to reproductive tract tissues. We identified numerous factors involved in GBS fitness in the FRT, including MtsA, which we confirmed contributes to GBS persistence in the vaginal tract. Our results further suggest that MtsA has a role in the maintenance of Mn^2+^ homeostasis and resistance to calprotectin-mediated metal chelation and contributes to the ability of GBS to withstand oxidative stress. MtsA is highly conserved across Gram-positive pathogens, is a component of the GBS core genome (67), and is essential for fitness in the female vaginal tract, thus warranting a more thorough understanding of its role in GBS physiology and suggesting its potential as a future therapeutic target.

## MATERIALS AND METHODS

### Bacterial strains and growth conditions.

Streptococcus agalactiae (GBS) isolates CJB111 (serotype V) and A909 (serotype Ia) and mutant strains CJB111 Δ*mtsA* and A909 Δ*mtsA* were cultured in Todd-Hewitt broth (THB) at 37°C. Mutant strains were constructed as previously described (30). Briefly, genomic 5′ and 3′ regions flanking the *mtsA* gene were amplified and fused with a spectinomycin cassette by FailSafe PCR (Lucigen). Fragments and pHY304 vector were digested with restriction enzymes XbaI and XmaI and ligated using a Quick Ligation kit (NEB). The ligation reaction product was transformed into chemically competent Escherichia coli MC1061 and selected on LB agar with ampicillin. pHY304 plasmids were purified from E. coli and electroporated into GBS CJB111 and A909 genetic backgrounds and selected on Todd-Hewitt agar (THA) plates with 100 μg/mL spectinomycin. The WT and mutant strains grew to the same optical density at stationary phase (~5 h) in nutrient-rich THB (see Fig. S5 in the supplemental material). The Δ*mtsA*p*mtsA* complemented strain (Δ*mtsA*+) was generated using the pDCERM expression vector, digested with XbaI and EcoRI-HF, and electroporated into GBS Δ*mtsA* strains, and mutants were selected on THA with erythromycin at 5 μg/mL. The complemented strain exhibited a significant growth defect during log phase (Fig. S5). All constructs were confirmed by PCR and sequencing. Primers used in the construction of the *mtsA* mutant and complemented strains are listed in Table S5.

10.1128/mbio.00985-22.5 FIG S5 Growth analysis of CJB111 WT, Δ*mtsA*, and Δ*mtsA*+ strains in THB. Growth was assessed by optical density (OD_600_) (A) or quantification of CFU/mL (B). Significance was determined by two-way ANOVA with Dunnett’s multiple-comparison test. *, *P < *0.05; **, *P < *0.01; ****, *P < *0.0001; ns, not significant. Download FIG S5, TIF file, 0.3 MB. Copyright © 2022 Burcham et al. 2022 Burcham et al. https://creativecommons.org/licenses/by/4.0/ This content is distributed under the terms of the Creative Commons Attribution 4.0 International license.

10.1128/mbio.00985-22.10 TABLE S5 Primer sequences used in this study. Download Table S5, DOCX file, 0.01 MB. Copyright © 2022 Burcham et al. 2022 Burcham et al. https://creativecommons.org/licenses/by/4.0/ This content is distributed under the terms of the Creative Commons Attribution 4.0 International license.

### *In vivo* transposon library screening during vaginal colonization.

All animal experiments were conducted under the approval of the Institutional Animal Care and Use Committee (approval no. 00316) at the University of Colorado Anschutz Medical Campus and performed using accepted veterinary standards. We utilized a murine model of vaginal colonization as previously described (4, 27), in which female CD-1 mice were synchronized on day −1 with 17β-estradiol by intraperitoneal injection. On day 0, triplicate overnight cultures of GBS pooled *Krmit* library were back-diluted into THB with kanamycin at 300 μg/mL and grown to OD_600_ 0.4. Cultures were normalized in triplicate to 1 × 10^7^ CFU/10 μL and inoculated by pipetting directly into the vaginal lumen of triplicate groups with 3 mice/group (*n *= 9 total). Swabs of the vaginal lumen were collected on days 1, 2, and 3, and on day 3 post-colonization, mice were euthanized and vaginal, cervical, and uterine tissues were harvested. Samples collected from daily swabs and tissue homogenates were used to quantify the GBS CFU burden over time, and the remainder of each sample was spread plated onto CHROMagar Strep B with 300 μg/mL kanamycin to collect recovered transposon insertion mutants. Bacterial growth from spread plates of daily swabs and tissues were collected and pooled into triplicate groups, and genomic DNA was extracted using a ZymoBiomics DNA miniprep kit (Zymo Research).

### Transposon library sequencing.

Library preparation and sequencing were performed as previously described (68) by the Microarray and Genomics Core at the University of Colorado Anschutz Medical Campus. Briefly, genomic DNA was sheared to approximately 340-bp fragments and processed through the Ovation ultralow V2 DNA-Seq library preparation kit (Tecan), and 9 ng of each library was used as a template for enrichment by PCR (16 cycles) for the transposon insertions using *Krmit*-specific (TCGTCGGCAGCGTCAGATGTGTATAAGAGACAGCCGGGGACTTATCATCCAACC) and Illumina P7 primers. The enriched PCR products were diluted 1:100, and 20 μL was used as a template for an indexing PCR (9 cycles) using the TruSeq P5 indexing and P7 primers. Sequencing was performed to obtain roughly 20 million reads per sample using an Illumina NovaSeq 6000 system in a 150-base paired-end format.

### *In vivo* Tn-seq bioinformatic analyses.

Analysis of transposon sequencing data was performed as previously described with minor modifications (26), including the use of the recently improved Streptococcus agalactiae CJB111 (CP063198.1) genome as a reference for these reads. A range of 6 to 61 million raw reads/sample (518 million in total) were queried for quality with FastQC (69), and of those, a range of 2.6 to 31 million reads/sample (255 million in total) were deemed suitable and mapped to the GBS CJB111 (CP063198_sRNA) genome. To identify *mariner* inverted terminal repeats (ITRs) and transposon insertion TA sites, a Bio::SeqIO-based script was written (bin/consolidate_reads.pl), and when the ITR to TA was observed on the negative strand, the sequence was reverse complemented to trim to the insertion TA. The number of observed insertion points on each strand was counted, before removal of the *mariner* ITR leading sequences with cutadapt (70). In a further attempt to verify that the samples were sufficiently robust, we used the TPP preprocessing tools provided by the TRANSIT package (29). This used bwa (71) rather than bowtie/hisat2 for mapping and generating wig files suitable for the downstream TN-seq TRANSIT analytical methods. Invocations and preprocessing methods were recorded in the markdown document, tnseq_transit.Rmd. The libraries were quantified with respect to relative coverage, similarity, and saturation with respect to available TA insertion points by using the bowtie-derived counts (72, 73). The resulting alignments were converted to sorted/compressed binary alignments (74) and counted (75) against the reference genome coding DNA sequence (CDS) and intergenic regions. Comparison and normalization of control (input) and experimental (output) libraries were performed using DESeq2. Many of the postprocessing tasks were handled via the hpgltools R package (76), and functions used are italicized and suffixed with “()”. Clustering of orthologous groups of proteins (COGs) were assigned using EggNOG 5.0.0 (77), and Venn diagrams were calculated and plotted using BxToolBox (https://www.bioinforx.com).

### *In vivo* RNA sequencing during vaginal colonization and bioinformatic analyses.

For collection of control RNA from GBS grown *in vitro*, GBS was grown in triplicate in THB to an OD_600_ of 0.4, pelleted, resuspended in 1 mL TRIzol reagent, and stored at −80°C. For collection of RNA from GBS *in vivo*, we utilized a murine model of vaginal colonization as previously described (4, 27), in which female CD-1 mice were synchronized on day −1 with 17β-estradiol by intraperitoneal injection, and the next day, mice were vaginally inoculated with 10^7^ CFU of GBS CJB111. On days 1 and 3 postinoculation, the vaginal lumen of mice was swabbed, and swabs were placed into 100 μL of TRIzol reagent (Thermo Fisher), vortexed to dissociate bacteria from swabs, and stored at −80°C. Swab samples from 6 mice were pooled, and bacteria were lysed by bead beating for 2 min (30-s intervals with 30 s of ice treatment between each bead-beating cycle) at maximum speed. RNA was isolated following the manufacturer’s protocol using a Direct-zol RNA miniprep plus kit (Zymo Research).

Total RNA samples were sequenced by Illumina sequencing at the Univeristy of Colorado Anschutz Medical Campus Genomics and Microarray Core. One hundred twenty micrograms of total RNA was depleted of rRNAs, and libraries underwent 9 cycles of PCR prior to 1× AMPure bead purification. Libraries were sequenced using the Illumina NovaSeq 6000 sequencing platform with 75-base single reads at 40 million reads/sample. Raw sequencing reads in fastq format were aligned and annotated to a file containing rRNA reads from the clinical group B streptococcal isolate CJB111 reference genome (CP063198_sRNA) using the following Qiagen CLC Genomics Workbench default settings (version 21.0.5): mismatch cost, 2; insertion and deletion cost, 3; and length and similarity fraction, 0.8. Unmapped genes were then mapped to the CJB111 reference genome (CP063198_sRNA) for RNA-seq analysis in CLC. Volcano plots for differentially expressed genes were generated using GraphPad Prism 9.0.

### ICP-MS.

Overnight cultures of GBS CJB111 WT, Δ*mtsA*, and Δ*mtsA*+ strains were back diluted into 10 mL fresh THB and were grown to an OD_600_ of 0.4. Bacterial cells were pelleted, rinsed in 1 mL nuclease-free H_2_O, pelleted again, and desiccated at 65°C for 1 h. For inductively coupled plasma mass spectrometry (ICP-MS), bacterial pellets were digested at 65°C overnight in 50 μL nitric acid. Samples were centrifuged and diluted 1:10 into an Agilent ICP-MS 7500cx autosampler. Mn^55^ concentrations in parts per billion (μg/mL) were determined by plotting samples against a standard curve and were normalized across bacterial strains by cell dry weight. The data are represented as the mean Mn^55^ concentration from four independent cultures of WT or Δ*mtsA* mutant or five independent cultures of GBS WT, Δ*mtsA*, and Δ*mtsA*+ strains.

### Calprotectin growth assays.

Briefly, GBS cultures grown overnight for 18 h in THB were diluted 1:50 into 100 μL in a 96-well microtiter plate with 38% 3× THB and 62% calprotectin buffer (20 mM Tris [pH 7.5], 100 mM NaCl, 3 mM CaCl_2_) and 0 to 960 μg/mL recombinant calprotectin (78, 79). The range of recombinant calprotectin was previously established for assaying bacterial survival *in vitro* (33, 54). At 6 h postinoculation, growth was assessed by measuring the optical density (OD_600_). In experiments where Mn^2+^ was supplemented to counter calprotectin chelation, MnCl_2_ was added at 10 μM.

### Quantitative reverse transcriptase PCR (qRT-PCR).

GBS were grown to mid-logarithmic phase (OD_600_, 0.4) in 3× THB medium plus calprotectin buffer and incubated with calprotectin at 960 μg/mL alone for 30 min or calprotectin at 960 μg/mL for 15 min followed by 50 μM MnCl_2_ supplementation for an additional 15 min. Following incubation, bacteria were centrifuged at 5,000 × *g* for 5 min, total RNA was extracted (Macherey-Nagel), and cDNA was synthesized (Quanta Biosciences) per the manufacturers’ instructions. Primers used in this study are listed in Table S5.

### Oxidative stress experiments.

Overnight cultures of GBS CJB111 WT and Δ*mtsA* were back-diluted 1:50 into fresh THB control medium or THB with added 0 to 0.01% H_2_O_2_ or 0.25 mM paraquat and were grown at 37°C for 6 h. Following incubation, optical density (OD_600_) was measured. All experiments were performed in technical triplicate wells and were repeated in three biologically independent experiments. The mean result for the technical replicate wells was calculated for each independent experiment, and the data shown are the mean calculated from all three biologically independent experiments.

### Statistical analyses.

Significance was determined using an unpaired two-tailed Student's *t* test for ICP-MS experiments and CD-1 tissue burden data, one-way analysis of variance (ANOVA) with Tukey’s multiple-comparison test for qRT-PCR experiments, two-way ANOVA with Tukey’s multiple-comparison test for calprotectin growth experiments, and two-way ANOVA with Sídák’s multiple-comparison test for colonization data, C57BL/6 and *S100A9^−/−^* tissue burden, and oxidative stress experiments. Statistical significance was accepted when the *P* value was <α, with α equal to 0.05. All statistical analyses were performed using GraphPad Prism 9.3.1.

### Data availability.

Sequencing reads from the transposon sequencing analyses are available in the NCBI Sequence Read Archive (SRA) under accession numbers PRJNA820592 (Tn-seq) and PRJNA821062 (RNA-seq).

## References

[1] Martin JA, Hamilton BE, Osterman MJ. 2018. Births in the United States. NCHS Data Brief no. 318. National Center for Health Statistics, Hyattsville, MD.

[2] Hoyert DL, Gregory EC. 2016. Cause of fetal death: data from the fetal death report, 2014. Natl Vital Stat Rep 65:1–25. 27805550

[3] Agrawal V, Hirsch E. 2012. Intrauterine infection and preterm labor. Semin Fetal Neonatal Med 17:12–19. doi:10.1016/j.siny.2011.09.001. 21944863 PMC3242863

[4] Patras KA, Rosler B, Thoman ML, Doran KS. 2015. Characterization of host immunity during persistent vaginal colonization by group B *Streptococcus*. Mucosal Immunol 8:1339–1348. doi:10.1038/mi.2015.23. 25850655 PMC4598252

[5] Armistead B, Oler E, Waldorf KA, Rajagopal L. 2019. The double life of group B Streptococcus: asymptomatic colonizer and potent pathogen. J Mol Biol 431:2914–2931. doi:10.1016/j.jmb.2019.01.035. 30711542 PMC6646060

[6] Akiyama K, Nishioka K, Khan KN, Tanaka Y, Mori T, Nakaya T, Kitawaki J. 2019. Molecular detection of microbial colonization in cervical mucus of women with and without endometriosis. Am J Reprod Immunol 82:e13147. doi:10.1111/aji.13147. 31087436

[7] Seale AC, Bianchi-Jassir F, Russell NJ, Kohli-Lynch M, Tann CJ, Hall J, Madrid L, Blencowe H, Cousens S, Baker CJ, Bartlett L, Cutland C, Gravett MG, Heath PT, Ip M, Le Doare K, Madhi SA, Rubens CE, Saha SK, Schrag SJ, Sobanjo-Ter Meulen A, Vekemans J, Lawn JE. 2017. Estimates of the burden of group B streptococcal disease worldwide for pregnant women, stillbirths, and children. Clin Infect Dis 65:S200–S219. doi:10.1093/cid/cix664. 29117332 PMC5849940

[8] Seale AC, Blencowe H, Bianchi-Jassir F, Embleton N, Bassat Q, Ordi J, Menendez C, Cutland C, Briner C, Berkley JA, Lawn JE, Baker CJ, Bartlett L, Gravett MG, Heath PT, Ip M, Le Doare K, Rubens CE, Saha SK, Schrag S, Meulen AS, Vekemans J, Madhi SA. 2017. Stillbirth with group B *Streptococcus* disease worldwide: systematic review and meta-analyses. Clin Infect Dis 65:S125–S132. doi:10.1093/cid/cix585. 29117322 PMC5850020

[9] Allen U, Nimrod C, Macdonald N, Toye B, Stephens D, Marchessault V. 1999. Relationship between antenatal group B streptococcal vaginal colonization and premature labour. Paediatr Child Health 4:465–469. doi:10.1093/pch/4.7.465. 20212961 PMC2827758

[10] Nandyal RR. 2008. Update on group B streptococcal infections: perinatal and neonatal periods. J Perinat Neonatal Nurs 22:230–237. doi:10.1097/01.JPN.0000333925.30328.fd. 18708876

[11] Campbell JR, Hillier SL, Krohn MA, Ferrieri P, Zaleznik DF, Baker CJ. 2000. Group B streptococcal colonization and serotype-specific immunity in pregnant women at delivery. Obstet Gynecol 96:498–503. doi:10.1016/s0029-7844(00)00977-7. 11004347

[12] Romero R, Gómez R, Chaiworapongsa T, Conoscenti G, Cheol Kim J, Mee Kim Y. 2001. The role of infection in preterm labour and delivery. Paediatr Perinat Epidemiol 15:41–56. doi:10.1046/j.1365-3016.2001.00007.x. 11520399

[13] Hall J, Adams NH, Bartlett L, Seale AC, Lamagni T, Bianchi-Jassir F, Lawn JE, Baker CJ, Cutland C, Heath PT, Ip M, Le Doare K, Madhi SA, Rubens CE, Saha SK, Schrag S, Sobanjo-Ter Meulen A, Vekemans J, Gravett MG. 2017. Maternal disease with group B *Streptococcus* and serotype distribution worldwide: systematic review and meta-analyses. Clin Infect Dis 65:S112–S124. doi:10.1093/cid/cix660. 29117328 PMC5850000

[14] Madrid L, Seale AC, Kohli-Lynch M, Edmond KM, Lawn JE, Heath PT, Madhi SA, Baker CJ, Bartlett L, Cutland C, Gravett MG, Ip M, Le Doare K, Rubens CE, Saha SK, Sobanjo-Ter Meulen A, Vekemans J, Schrag S, Infant GBS Disease Investigator Group. 2017. Infant group B streptococcal disease incidence and serotypes worldwide: systematic review and meta-analyses. Clin Infect Dis 65:S160–S172. doi:10.1093/cid/cix656. 29117326 PMC5850457

[15] Melin P. 2011. Neonatal group B streptococcal disease: from pathogenesis to preventive strategies. Clin Microbiol Infect 17:1294–1303. doi:10.1111/j.1469-0691.2011.03576.x. 21672083

[16] Cain AK, Barquist L, Goodman AL, Paulsen IT, Parkhill J, van Opijnen T. 2020. A decade of advances in transposon-insertion sequencing. Nat Rev Genet 21:526–540. doi:10.1038/s41576-020-0244-x. 32533119 PMC7291929

[17] Rowe HM, Karlsson E, Echlin H, Chang T-C, Wang L, van Opijnen T, Pounds SB, Schultz-Cherry S, Rosch JW. 2019. Bacterial factors required for transmission of *Streptococcus pneumoniae* in mammalian hosts. Cell Host Microbe 25:884–891.e6. doi:10.1016/j.chom.2019.04.012. 31126758 PMC6598203

[18] Arenas J, Zomer A, Harders-Westerveen J, Bootsma HJ, De Jonge MI, Stockhofe-Zurwieden N, Smith HE, De Greeff A. 2020. Identification of conditionally essential genes for *Streptococcus suis* infection in pigs. Virulence 11:446–464. doi:10.1080/21505594.2020.1764173. 32419603 PMC7239030

[19] Shields RC, Zeng L, Culp DJ, Burne RA. 2018. Genomewide identification of essential genes and fitness determinants of *Streptococcus mutans* UA159. mSphere 3:e00031-18. doi:10.1128/mSphere.00031-18. 29435491 PMC5806208

[20] Hooven TA, Catomeris AJ, Akabas LH, Randis TM, Maskell DJ, Peters SE, Ott S, Santana-Cruz I, Tallon LJ, Tettelin H, Ratner AJ. 2016. The essential genome of *Streptococcus agalactiae*. BMC Genomics 17:406. doi:10.1186/s12864-016-2741-z. 27229469 PMC4881062

[21] Hooven TA, Catomeris AJ, Bonakdar M, Tallon LJ, Santana-Cruz I, Ott S, Daugherty SC, Tettelin H, Ratner AJ. 2018. The *Streptococcus agalactiae* stringent response enhances virulence and persistence in human blood. Infect Immun 86:e00612-17. doi:10.1128/IAI.00612-17. 29109175 PMC5736797

[22] Matthews AJ, Rowe HM, Rosch JW, Camilli A. 2021. A Tn-seq screen of *Streptococcus pneumoniae* uncovers DNA repair as the major pathway for desiccation tolerance and transmission. Infect Immun 89:e00713-20. doi:10.1128/IAI.00713-20. PMC8281258 34031124

[23] van Opijnen T, Lazinski DW, Camilli A. 2014. Genome‐wide fitness and genetic interactions determined by Tn‐seq, a high‐throughput massively parallel sequencing method for microorganisms. Cur Protoc Mol Biol 106:24. doi:10.1002/0471142727.mb0716s106. PMC4568079 24733243

[24] Le Breton Y, Belew AT, Valdes KM, Islam E, Curry P, Tettelin H, Shirtliff ME, El-Sayed NM, McIver KS. 2015. Essential genes in the core genome of the human pathogen *Streptococcus pyogenes*. Sci Rep 5:9838–9813. doi:10.1038/srep09838. 25996237 PMC4440532

[25] Le Breton Y, Belew AT, Freiberg JA, Sundar GS, Islam E, Lieberman J, Shirtliff ME, Tettelin H, El-Sayed NM, McIver KS. 2017. Genome-wide discovery of novel M1T1 group A streptococcal determinants important for fitness and virulence during soft-tissue infection. PLoS Pathog 13:e1006584. doi:10.1371/journal.ppat.1006584. 28832676 PMC5584981

[26] Burcham LR, Breton YL, Radin JN, Spencer BL, Deng L, Hiron A, Ransom MR, Mendonça JC, Belew AT, El-Sayed NM, McIver KS, Kehl-Fie TE, Doran KS, Cook L, McDaniel LS. 2020. Identification of zinc-dependent mechanisms used by group B *Streptococcus* to overcome calprotectin-mediated stress. mBio 11:e02302-20. doi:10.1128/mBio.02302-20. 33173000 PMC7667036

[27] Patras KA, Doran KS. 2016. A murine model of group B *Streptococcus* vaginal colonization. J Vis Exp 2016:e54708. doi:10.3791/54708. PMC5226234 27911391

[28] Spencer BL, Chatterjee A, Duerkop BA, Baker CJ, Doran KS, Hotopp JCD. 2021. Complete genome sequence of neonatal clinical group B streptococcal isolate CJB111. Microbiol Resour Announc 10:e01268-20. doi:10.1128/MRA.01268-20. 33446593 PMC7849706

[29] DeJesus MA, Ambadipudi C, Baker R, Sassetti C, Ioerger TR. 2015. TRANSIT—a software tool for Himar1 TnSeq analysis. PLoS Comput Biol 11:e1004401. doi:10.1371/journal.pcbi.1004401. 26447887 PMC4598096

[30] Spencer BL, Tak U, Mendonça JC, Nagao PE, Niederweis M, Doran KS. 2021. A type VII secretion system in group B Streptococcus mediates cytotoxicity and virulence. PLoS Pathog 17:e1010121. doi:10.1371/journal.ppat.1010121. 34871327 PMC8675928

[31] Cook LCC, Hu H, Maienschein-Cline M, Federle MJ. 2018. A vaginal tract signal detected by the group B *Streptococcus* SaeRS system elicits transcriptomic changes and enhances murine colonization. Infect Immun 86:e00762-17. doi:10.1128/IAI.00762-17. 29378799 PMC5865029

[32] Hayden JA, Brophy MB, Cunden LS, Nolan EM. 2013. High-affinity manganese coordination by human calprotectin is calcium-dependent and requires the histidine-rich site formed at the dimer interface. J Am Chem Soc 135:775–787. doi:10.1021/ja3096416. 23276281 PMC3575579

[33] Kehl-Fie TE, Chitayat S, Hood MI, Damo S, Restrepo N, Garcia C, Munro KA, Chazin WJ, Skaar EP. 2011. Nutrient metal sequestration by calprotectin inhibits bacterial superoxide defense, enhancing neutrophil killing of *Staphylococcus aureus*. Cell Host Microbe 10:158–164. doi:10.1016/j.chom.2011.07.004. 21843872 PMC3157011

[34] Damo SM, Kehl-Fie TE, Sugitani N, Holt ME, Rathi S, Murphy WJ, Zhang Y, Betz C, Hench L, Fritz G, Skaar EP, Chazin WJ. 2013. Molecular basis for manganese sequestration by calprotectin and roles in the innate immune response to invading bacterial pathogens. Proc Natl Acad Sci USA 110:3841–3846. doi:10.1073/pnas.1220341110. 23431180 PMC3593839

[35] Diaz-Ochoa VE, Lam D, Lee CS, Klaus S, Behnsen J, Liu JZ, Chim N, Nuccio S-P, Rathi SG, Mastroianni JR, Edwards RA, Jacobo CM, Cerasi M, Battistoni A, Ouellette AJ, Goulding CW, Chazin WJ, Skaar EP, Raffatellu M. 2016. *Salmonella* mitigates oxidative stress and thrives in the inflamed gut by evading calprotectin-mediated manganese sequestration. Cell Host Microbe 19:814–825. doi:10.1016/j.chom.2016.05.005. 27281571 PMC4901528

[36] Aguirre JD, Culotta VC. 2012. Battles with iron: manganese in oxidative stress protection. J Biol Chem 287:13541–13548. doi:10.1074/jbc.R111.312181. 22247543 PMC3340200

[37] Faralla C, Metruccio MM, Chiara MD, Mu R, Patras KA, Muzzi A, Grandi G, Margarit I, Doran KS, Janulczyk R, Nizet V, McDaniel LS. 2014. Analysis of two-component systems in group B *Streptococcus* shows that RgfAC and the novel FspSR modulate virulence and bacterial fitness. mBio 5:e00870-14. doi:10.1128/mBio.00870-14. 24846378 PMC4030450

[38] Deng L, Mu R, Weston TA, Spencer BL, Liles RP, Doran KS, Freitag NE. 2018. Characterization of a two-component system transcriptional regulator, LtdR, that impacts group B streptococcal colonization and disease. Infect Immun 86:e00822-17. doi:10.1128/IAI.00822-17. 29685987 PMC6013667

[39] Sheen TR, Jimenez A, Wang NY, Banerjee A, van Sorge NM, Doran KS. 2011. Serine-rich repeat proteins and pili promote *Streptococcus agalactiae* colonization of the vaginal tract. J Bacteriol 193:6834–6842. doi:10.1128/JB.00094-11. 21984789 PMC3232834

[40] Jiang S, Park SE, Yadav P, Paoletti LC, Wessels MR. 2012. Regulation and function of pilus island 1 in group B *Streptococcus*. J Bacteriol 194:2479–2490. doi:10.1128/JB.00202-12. 22408160 PMC3347183

[41] Kolar SL, Kyme P, Tseng CW, Soliman A, Kaplan A, Liang J, Nizet V, Jiang D, Murali R, Arditi M, Underhill DM, Liu GY. 2015. Group B *Streptococcus* evades host immunity by degrading hyaluronan. Cell Host Microbe 18:694–704. doi:10.1016/j.chom.2015.11.001. 26651945 PMC4683412

[42] Coleman M, Armistead B, Orvis A, Quach P, Brokaw A, Gendrin C, Sharma K, Ogle J, Merillat S, Dacanay M, Wu T-Y, Munson J, Baldessari A, Vornhagen J, Furuta A, Nguyen S, Waldorf KMA, Rajagopal L, Coyne CB. 2021. Hyaluronidase impairs neutrophil function and promotes group B *Streptococcus* invasion and preterm labor in nonhuman primates. mBio 12:e03115-20. doi:10.1128/mBio.03115-20. 33402537 PMC8545101

[43] Patras KA, Wang N-Y, Fletcher EM, Cavaco CK, Jimenez A, Garg M, Fierer J, Sheen TR, Rajagopal L, Doran KS. 2013. Group B streptococcus CovR regulation modulates host immune signalling pathways to promote vaginal colonization. Cell Microbiol 15:1154–1167. doi:10.1111/cmi.12105. 23298320 PMC3657335

[44] Royet K, Parisot N, Rodrigue A, Gueguen E, Condemine G. 2019. Identification by Tn‐seq of Dickeya dadantii genes required for survival in chicory plants. Mol Plant Pathol 20:287–306. doi:10.1111/mpp.12754. 30267562 PMC6637903

[45] Fabian BK, Tetu SG, Paulsen IT. 2020. Application of transposon insertion sequencing to agricultural science. Front Plant Sci 11:291. doi:10.3389/fpls.2020.00291. 32256512 PMC7093568

[46] Moulin P, Patron K, Cano C, Zorgani MA, Camiade E, Borezée-Durant E, Rosenau A, Mereghetti L, Hiron A. 2016. The Adc/Lmb system mediates zinc acquisition in *Streptococcus agalactiae* and contributes to bacterial growth and survival. J Bacteriol 198:3265–3277. doi:10.1128/JB.00614-16. 27672194 PMC5116930

[47] Moulin P, Rong V, Ribeiro E Silva A, Pederick VG, Camiade E, Mereghetti L, McDevitt CA, Hiron A. 2019. Defining the role of the *Streptococcus agalactiae* Sht-family proteins in zinc acquisition and complement evasion. J Bacteriol 201:e00757-18. doi:10.1128/JB.00757-18. 30745371 PMC6436357

[48] Cobine P, Wickramasinghe WA, Harrison MD, Weber T, Solioz M, Dameron CT. 1999. The *Enterococcus hirae* copper chaperone CopZ delivers copper(I) to the CopY repressor. FEBS Lett 445:27–30. doi:10.1016/S0014-5793(99)00091-5. 10069368

[49] Sullivan MJ, Goh KGK, Gosling D, Katupitiya L, Ulett GC, Henkin TM. 2021. Copper intoxication in group B *Streptococcus* triggers transcriptional activation of the *cop* operon that contributes to enhanced virulence during acute infection. J Bacteriol 203:e00315-21. doi:10.1128/JB.00315-21. PMC8447484 34251869

[50] Glauninger H, Zhang Y, Higgins KA, Jacobs AD, Martin JE, Fu Y, Coyne Rd HJ, Bruce KE, Maroney MJ, Clemmer DE, Capdevila DA, Giedroc DP. 2018. Metal-dependent allosteric activation and inhibition on the same molecular scaffold: the copper sensor CopY from *Streptococcus pneumoniae*. Chem Sci 9:105–118. doi:10.1039/c7sc04396a. 29399317 PMC5772342

[51] Johnson MDL, Kehl-Fie TE, Klein R, Kelly J, Burnham C, Mann B, Rosch JW, Camilli A. 2015. Role of copper efflux in pneumococcal pathogenesis and resistance to macrophage-mediated immune clearance. Infect Immun 83:1684–1694. doi:10.1128/IAI.03015-14. 25667262 PMC4363445

[52] Neubert MJ, Dahlmann EA, Ambrose A, Johnson MDL, Sawers G. 2017. Copper chaperone CupA and zinc control CopY regulation of the pneumococcal *cop* operon. mSphere 2:e00372-17. doi:10.1128/mSphere.00372-17. PMC5646241 29062896

[53] Kehres DG, Zaharik ML, Finlay BB, Maguire ME. 2000. The NRAMP proteins of *Salmonella typhimurium* and *Escherichia coli* are selective manganese transporters involved in the response to reactive oxygen. Mol Microbiol 36:1085–1100. doi:10.1046/j.1365-2958.2000.01922.x. 10844693

[54] Kehl-Fie TE, Zhang Y, Moore JL, Farrand AJ, Hood MI, Rathi S, Chazin WJ, Caprioli RM, Skaar EP. 2013. MntABC and MntH contribute to systemic *Staphylococcus aureus* infection by competing with calprotectin for nutrient manganese. Infect Immun 81:3395–3405. doi:10.1128/IAI.00420-13. 23817615 PMC3754211

[55] Shabayek S, Bauer R, Mauerer S, Mizaikoff B, Spellerberg B. 2016. A streptococcal NRAMP homologue is crucial for the survival of *Streptococcus agalactiae* under low pH conditions. Mol Microbiol 100:589–606. doi:10.1111/mmi.13335. 27150893

[56] Eijkelkamp BA, McDevitt CA, Kitten T. 2015. Manganese uptake and streptococcal virulence. Biometals 28:491–508. doi:10.1007/s10534-015-9826-z. 25652937 PMC5800397

[57] Rosch JW, Gao G, Ridout G, Wang YD, Tuomanen EI. 2009. Role of the manganese efflux system mntE for signalling and pathogenesis in *Streptococcus pneumoniae*. Mol Microbiol 72:12–25. doi:10.1111/j.1365-2958.2009.06638.x. 19226324 PMC2706702

[58] Janulczyk R, Ricci S, Björck L. 2003. MtsABC is important for manganese and iron transport, oxidative stress resistance, and virulence of *Streptococcus pyogenes*. Infect Immun 71:2656–2664. doi:10.1128/IAI.71.5.2656-2664.2003. 12704140 PMC153223

[59] Corbin BD, Seeley EH, Raab A, Feldmann J, Miller MR, Torres VJ, Anderson KL, Dattilo BM, Dunman PM, Gerads R, Caprioli RM, Nacken W, Chazin WJ, Skaar EP. 2008. Metal chelation and inhibition of bacterial growth in tissue abscesses. Science 319:962–965. doi:10.1126/science.1152449. 18276893

[60] Weinberg ED. 1975. Nutritional immunity: host's attempt to withhold iron from microbial invaders. JAMA 231:39–41. doi:10.1001/jama.1975.03240130021018. 1243565

[61] Nakashige TG, Zhang B, Krebs C, Nolan EM. 2015. Human calprotectin is an iron-sequestering host-defense protein. Nat Chem Biol 11:765–771. doi:10.1038/nchembio.1891. 26302479 PMC4575267

[62] Obisesan AO, Zygiel EM, Nolan EM. 2021. Bacterial responses to iron withholding by calprotectin. Biochemistry 60:3337–3346. doi:10.1021/acs.biochem.1c00572. 34739212 PMC8595822

[63] Zygiel EM, Nelson CE, Brewer LK, Oglesby-Sherrouse AG, Nolan EM. 2019. The human innate immune protein calprotectin induces iron starvation responses in *Pseudomonas aeruginosa*. J Biol Chem 294:3549–3562. doi:10.1074/jbc.RA118.006819. 30622135 PMC6416435

[64] Bray BA, Sutcliffe IC, Harrington DJ. 2009. Expression of the MtsA lipoprotein of *Streptococcus agalactiae* A909 is regulated by manganese and iron. Antonie Van Leeuwenhoek 95:101–109. doi:10.1007/s10482-008-9291-6. 18982279

[65] Papp-Wallace KM, Maguire ME. 2006. Manganese transport and the role of manganese in virulence. Annu Rev Microbiol 60:187–209. doi:10.1146/annurev.micro.60.080805.142149. 16704341

[66] Poyart C, Pellegrini E, Gaillot O, Boumaila C, Baptista M, Trieu-Cuot P. 2001. Contribution of Mn-cofactored superoxide dismutase (SodA) to the virulence of *Streptococcus agalactiae*. Infect Immun 69:5098–5106. doi:10.1128/IAI.69.8.5098-5106.2001. 11447191 PMC98605

[67] Tettelin H, Masignani V, Cieslewicz MJ, Donati C, Medini D, Ward NL, Angiuoli SV, Crabtree J, Jones AL, Durkin AS, DeBoy RT, Davidsen TM, Mora M, Scarselli M, Margarit y Ros I, Peterson JD, Hauser CR, Sundaram JP, Nelson WC, Madupu R, Brinkac LM, Dodson RJ, Rosovitz MJ, Sullivan SA, Daugherty SC, Haft DH, Selengut J, Gwinn ML, Zhou L, Zafar N, Khouri H, Radune D, Dimitrov G, Watkins K, O'Connor KJB, Smith S, Utterback TR, White O, Rubens CE, Grandi G, Madoff LC, Kasper DL, Telford JL, Wessels MR, Rappuoli R, Fraser CM. 2005. Genome analysis of multiple pathogenic isolates of *Streptococcus agalactiae*: implications for the microbial “pan-genome.” Proc Natl Acad Sci USA 102:13950–13955. doi:10.1073/pnas.0506758102. 16172379 PMC1216834

[68] Dale JL, Beckman KB, Willett JLE, Nilson JL, Palani NP, Baller JA, Hauge A, Gohl DM, Erickson R, Manias DA, Sadowsky MJ, Dunny GM. 2018. Comprehensive functional analysis of the *Enterococcus faecalis* core genome using an ordered, sequence-defined collection of insertional mutations in strain OG1RF. mSystems 3:e00062-18. doi:10.1128/mSystems.00062-18. 30225373 PMC6134198

[69] Andrews S. 2010. FastQC: a quality control tool for high throughput sequence data. Babraham Bioinformatics, Babraham Institute, Cambridge, United Kingdom.

[70] Martin M. 2011. Cutadapt removes adapter sequences from high-throughput sequencing reads. EMBnet J 17:10–12. doi:10.14806/ej.17.1.200.

[71] Li M, Du X, Villaruz AE, Diep BA, Wang D, Song Y, Tian Y, Hu J, Yu F, Lu Y, Otto M. 2012. MRSA epidemic linked to a quickly spreading colonization and virulence determinant. Nat Med 18:816–819. doi:10.1038/nm.2692. 22522561 PMC3378817

[72] Langmead B. 2010. Aligning short sequencing reads with Bowtie. Curr Protoc Bioinformatics Chapter 11:Unit 11.17. doi:10.1002/0471250953.bi1107s32. PMC3010897 21154709

[73] Langmead B, Trapnell C, Pop M, Salzberg SL. 2009. Ultrafast and memory-efficient alignment of short DNA sequences to the human genome. Genome Biol 10:R25. doi:10.1186/gb-2009-10-3-r25. 19261174 PMC2690996

[74] Li H, Handsaker B, Wysoker A, Fennell T, Ruan J, Homer N, Marth G, Abecasis G, Durbin R, 1000 Genome Project Data Processing Subgroup. 2009. The sequence alignment/map format and SAMtools. Bioinformatics 25:2078–2079. doi:10.1093/bioinformatics/btp352. 19505943 PMC2723002

[75] Anders S, Pyl PT, Huber W. 2015. HTSeq—a Python framework to work with high-throughput sequencing data. bioinformatics, 31(2):166–169. 25260700 10.1093/bioinformatics/btu638 PMC4287950

[76] hpgltools. 2019. hpgltools. https://github.com/elsayed-lab/hpgltools. Accessed Feb 2021.

[77] Huerta-Cepas J, Szklarczyk D, Heller D, Hernández-Plaza A, Forslund SK, Cook H, Mende DR, Letunic I, Rattei T, Jensen Lars J, von Mering C, Bork P. 2019. eggNOG 5.0: a hierarchical, functionally and phylogenetically annotated orthology resource based on 5090 organisms and 2502 viruses. Nucleic Acids Res 47:D309–D314. doi:10.1093/nar/gky1085. 30418610 PMC6324079

[78] Grim KP, San Francisco B, Radin JN, Brazel EB, Kelliher JL, Párraga Solórzano PK, Kim PC, McDevitt CA, Kehl-Fie TE. 2017. The metallophore staphylopine enables *Staphylococcus aureus* to compete with the host for zinc and overcome nutritional immunity. mBio 8:e01281-17. doi:10.1128/mBio.01281-17. 29089427 PMC5666155

[79] Radin JN, Zhu J, Brazel EB, McDevitt CA, Kehl-Fie TE. 2019. Synergy between nutritional immunity and independent host defenses contributes to the importance of the MntABC manganese transporter during *Staphylococcus aureus* infection. Infect Immun 87:e00642-17. doi:10.1128/IAI.00642-18. 30348827 PMC6300641

[80] Burcham LR, Bath JR, Werlang CA, Lyon LM, Liu N, Evans C, Ribbeck K, Doran KS. 2022. Role of MUC5B during Group B Streptococcal Vaginal Colonization. Mbio 24;13(2):e00039-22. doi:10.1128/mbio.00039-22. PMC9040740 35323039

