# Supplementary figures and images for: Genomic Analyses Identify Manganese Homeostasis as a Driver of Group B Streptococcal Vaginal Colonization
Source: mBio. 2022 Jun 6;13(3):e00985-22. doi: 10.1128/mbio.00985-22 (PMC9239048; doi:10.1128/mbio.00985-22)

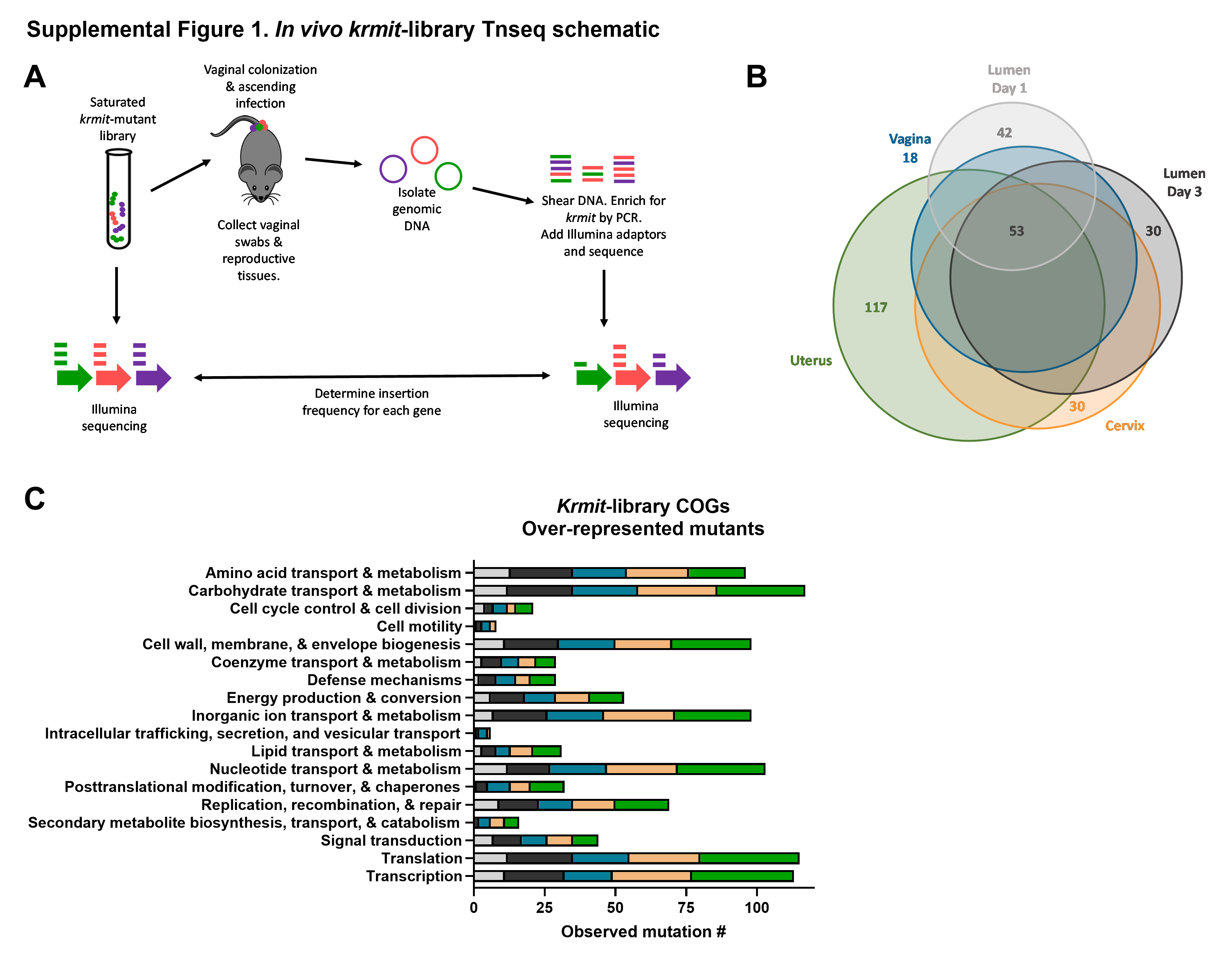

Supplement: FIG S1 [file mbio.00985-22-s0001.tif]

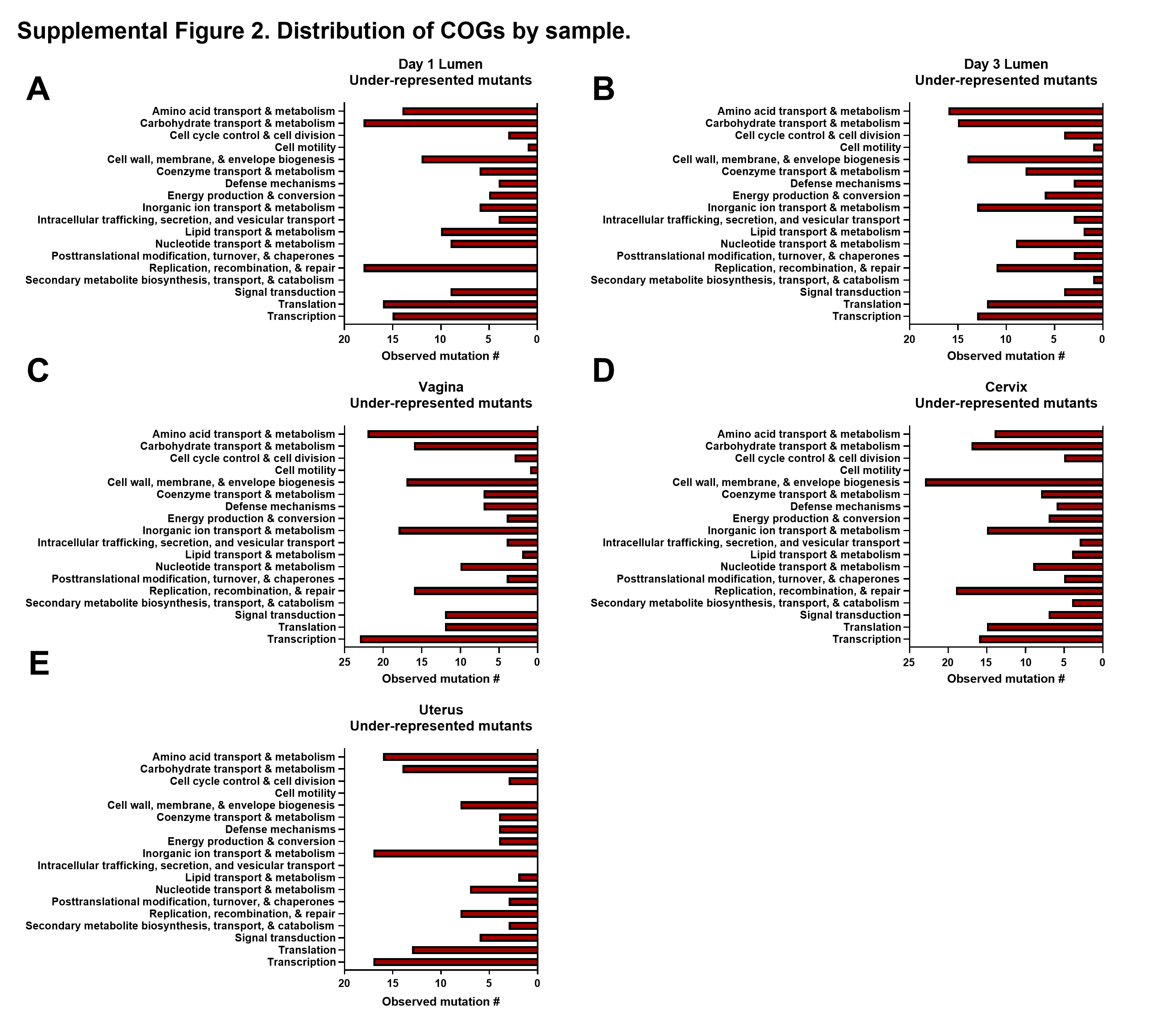

Supplement: FIG S2 [file mbio.00985-22-s0002.tif]

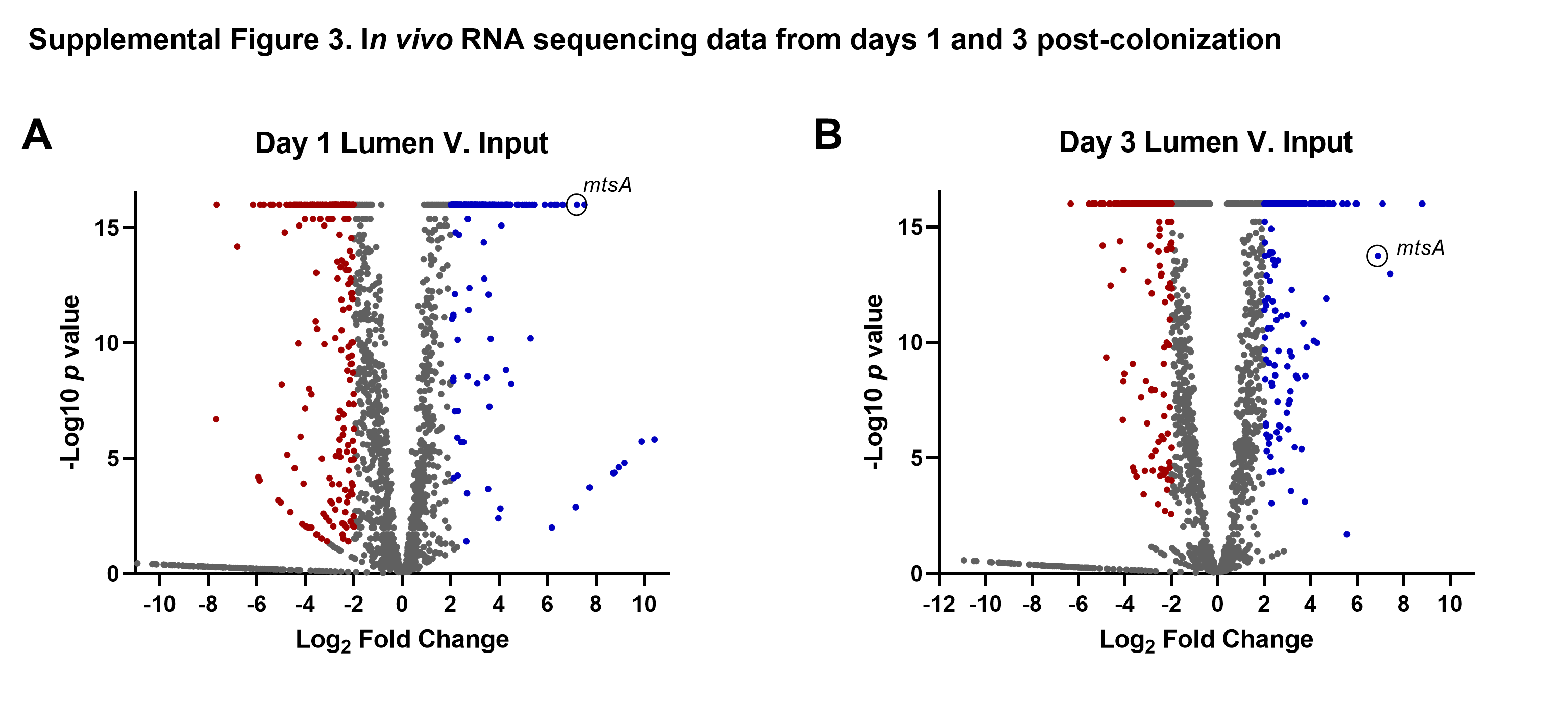

Supplement: FIG S3 [file mbio.00985-22-s0003.tif]

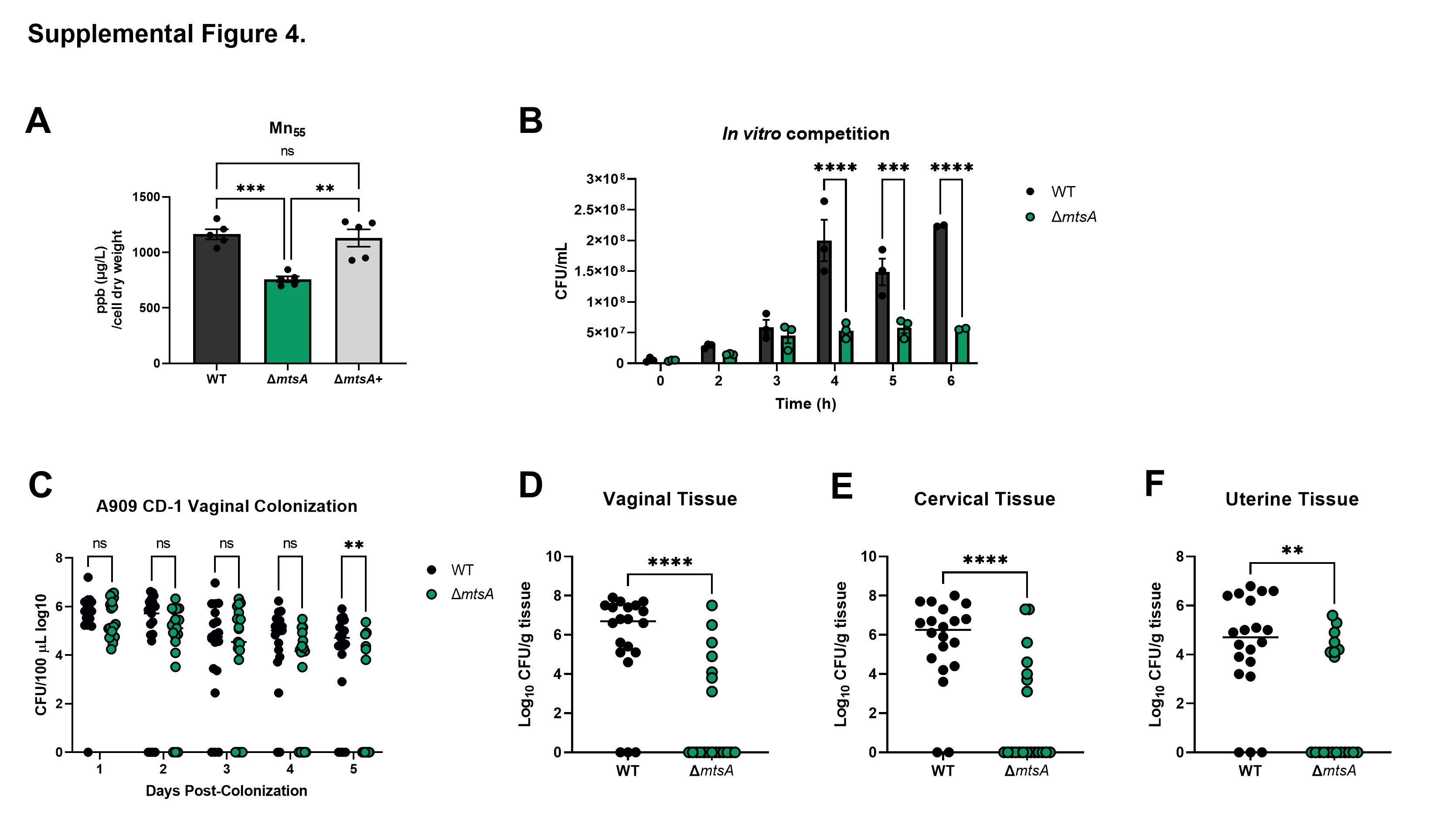

Supplement: FIG S4 [file mbio.00985-22-s0004.tif]

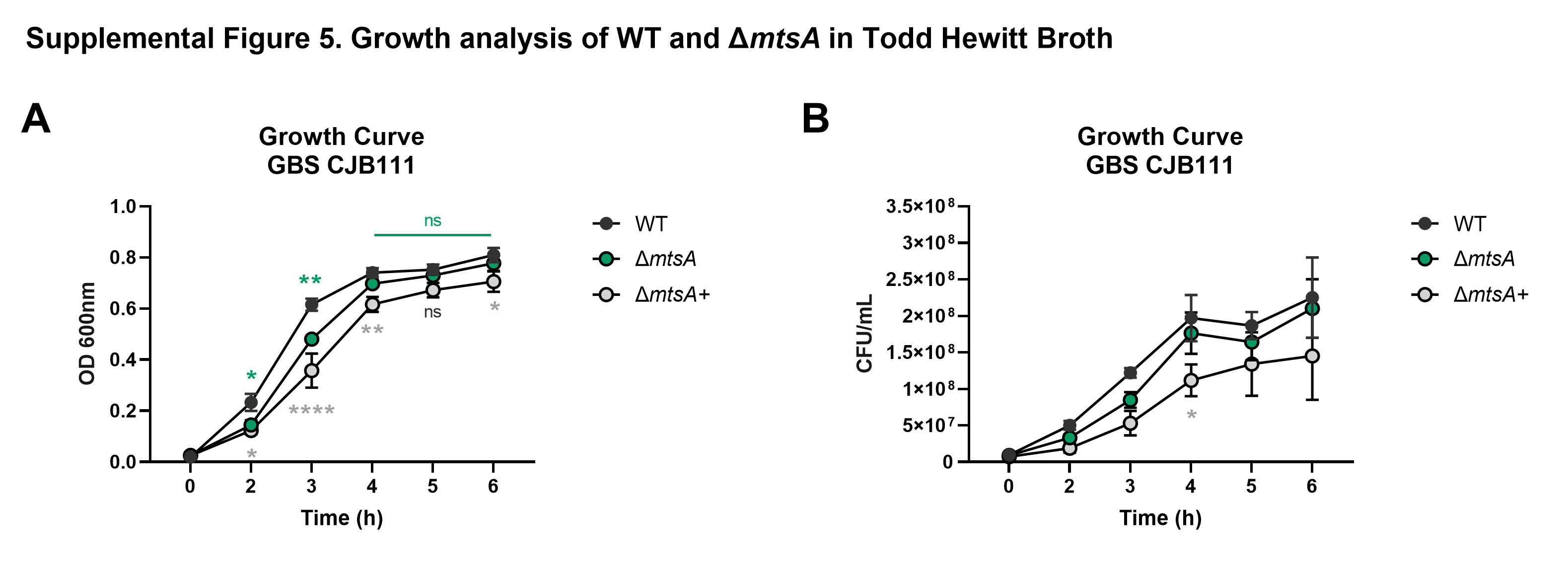

Supplement: FIG S5 [file mbio.00985-22-s0005.tif]
