# Supplementary material for: Genomic Analyses Identify Manganese Homeostasis as a Driver of Group B Streptococcal Vaginal Colonization
Source: mBio. 2022 Jun 6;13(3):e00985-22. doi: 10.1128/mbio.00985-22 (PMC9239048; doi:10.1128/mbio.00985-22)
Supplement: TABLE S2 [file mbio.00985-22-s0007.docx]

|  |  |  | **Fold change in reproductive tract** | | | | |
| --- | --- | --- | --- | --- | --- | --- | --- |
| **Representation**  **and gene number**  **(CJB111 genome)** | **Gene name** | **Description** | **D1** | **D3** | **VG** | **CX** | **UT** |
| **Under-represented in all samples** | | | | | | | |
| ID870_07885 |  | amino acid ABC transporter ATP-binding protein | -5.2 | -8.843 | -12.51 | -10.59 | -11.97 |
| ID870_01975 |  | VOC family protein | -2.142 | -7.759 | -6.219 | -9.887 | -6.105 |
| ID870_02480 |  | transporter substrate-binding domain-containing protein | -3.743 | -7.054 | -10.51 | -11 | -12.24 |
| ID870_07535 |  | PTS mannose/fructose/sorbose transporter family subunit IID | -3.947 | -6.863 | -8.249 | -10.86 | -30.53 |
| ID870_09355 |  | YhcH/YjgK/YiaL family protein | -3.835 | -10.11 | -12.89 | -12.27 | -13.51 |
| ID870_01925 |  | CotH kinase family protein | -2.587 | -4.708 | -7.717 | -5.548 | -7.338 |
| ID870_05505 |  | SDR family oxidoreductase | -9.264 | -13.6 | -17.27 | -19.96 | -18.41 |
| ID870_05310 |  | GntP family permease | -7.492 | -12.48 | -14.67 | -12.89 | -9.417 |
| ID870_04955 | *murA* | UDP-N-acetylglucosamine 1-carboxyvinyltransferase | -6.454 | -6.856 | -9.773 | -10.7 | -10.04 |
| ID870_07395 | *copZ* | heavy-metal-associated domain-containing protein | -9.538 | -16.55 | -18.27 | -31.92 | -40.23 |
| ID870_02010 | *mtsA* | metal ABC transporter substrate-binding protein | -4.948 | -6.711 | -10.29 | -9.193 | -7.443 |
| ID870_02665 | *fhuB* | iron ABC transporter permease | -2.888 | -6.308 | -9.129 | -8.375 | -7.943 |
| ID870_01765 | *trkA* | Trk system potassium transporter | -1.942 | -5.356 | -8.371 | -5.836 | -5.509 |
| ID870_02675 |  | ABC transporter ATP-binding protein | -2.534 | -5.254 | -8.06 | -5.698 | -4.935 |
| ID870_05940 | *fabZ* | 3-hydroxyacyl-ACP dehydratase | -3.191 | -8.334 | -7.566 | -7.557 | -6.845 |
| ID870_09420 |  | phosphoribosylaminoimidazolesuccinocarboxamide synthase | -8.77 | -12.47 | -37.71 | -19.93 | -16.21 |
| ID870_02135 |  | NUDIX domain-containing protein | -2.604 | -5.947 | -6.688 | -5.615 | -5.201 |
| ID870_06215 | *dcm* | DNA (cytosine-5-)-methyltransferase | -11.45 | -8.878 | -42.99 | -26.26 | -40.54 |
| ID870_06285 |  | DNA polymerase | -2.208 | -4.345 | -7.411 | -4.627 | -4.409 |
| ID870_09925 |  | helix-turn-helix domain-containing protein | -6.727 | -14.03 | -31.79 | -15.21 | -14.81 |
| ID870_06770 |  | ArgR family transcriptional regulator | -8.117 | -12.14 | -18.06 | -17.19 | -11.94 |
| ID870_05990 |  | hypothetical protein | -2.811 | -7.905 | -7.119 | -5.725 | -7.592 |
| ID870_05775 |  | FadR family transcriptional regulator | -6.216 | -8.421 | -10.78 | -9.333 | -13.07 |
| ID870_09255 | *rpsJ* | 30S ribosomal protein S10 | -7.903 | -19.45 | -39.37 | -22.7 | -39.37 |
| ID870_10165 | *rpmF* | 50S ribosomal protein L32 | -6.72 | -6.665 | -40.93 | -39.63 | -37.14 |
| ID870_08005 | *glyQ* | glycine--tRNA ligase subunit alpha | -6.376 | -8.699 | -10.95 | -9.475 | -9.352 |
| ID870_05630 |  | TIGR03943 family protein | -2.003 | -6.742 | -11.89 | -8.905 | -5.263 |
| ID870_08375 |  | NYN domain-containing protein | -2.84 | -7.102 | -7.976 | -10.55 | -5.439 |
| ID870_05205 |  | DUF3169 family protein | -3.355 | -7.012 | -8.893 | -6.675 | -7.509 |
| ID870_07150 |  | cupin | -3.286 | -8.676 | -11.51 | -11.62 | -14.34 |
| ID870_03180 |  | hypothetical protein | -2.298 | -5.357 | -7.322 | -3.984 | -5.863 |
| ID870_06210 |  | DUF4314 domain-containing protein | -11.95 | -9.856 | -11.71 | -12.53 | -10.1 |
| ID870_02115 |  | YozE family protein | -7.592 | -6.93 | -41.17 | -24.29 | -21.55 |
| ID870_01335 |  | aldo/keto reductase | -9.483 | -12.43 | -19.27 | -13.37 | -16.03 |
| ID870_02655 |  | DUF1803 domain-containing protein | -11.03 | -8.227 | -11.22 | -10.77 | -8.111 |
| ID870_04960 |  | DUF1146 domain-containing protein | -8.246 | -15.47 | -17.44 | -30.87 | -36.03 |
| ID870_06525 |  | NINE protein | -9.719 | -12.73 | -17.02 | -15.59 | -15.31 |
| ID870_00930 |  | YbaB/EbfC family nucleoid-associated protein | -4.574 | -6.749 | -11.02 | -8.368 | -11.63 |
| ID870_08775 |  | hypothetical protein | -9.461 | -8.047 | -41.1 | -41.1 | -21.43 |
| ID870_06185 |  | hypothetical protein | -10.84 | -10.56 | -16.77 | -13 | -15.9 |
| ID870_07635 |  | tRNA-Asn | -10.6 | -10.96 | -11.49 | -10.97 | -11.44 |
| ID870_00025 |  | hypothetical protein | -5.956 | -10.72 | -34.9 | -14.48 | -14.86 |
| ID870_04575 |  | hypothetical protein | -5.8 | -8.768 | -13.71 | -9.933 | -9.177 |
| ID870_03625 |  | tRNA-Arg | -7.053 | -17.17 | -37.23 | -40.95 | -33.24 |
| ID870_09990 |  | hypothetical protein | -9.002 | -12.78 | -13.72 | -15.63 | -43.12 |
| ID870_02305 |  | hypothetical protein | -3.778 | -9.721 | -10.15 | -9.207 | -21.97 |
| ID870_06090 |  | hypothetical protein | -4.207 | -6.034 | -9.273 | -8.385 | -9.372 |
