# Supplementary material for: Genomic Analyses Identify Manganese Homeostasis as a Driver of Group B Streptococcal Vaginal Colonization
Source: mBio. 2022 Jun 6;13(3):e00985-22. doi: 10.1128/mbio.00985-22 (PMC9239048; doi:10.1128/mbio.00985-22)
Supplement: TABLE S3 [file mbio.00985-22-s0008.docx]

|  |  |  | **Fold change in reproductive tract** | | | | |
| --- | --- | --- | --- | --- | --- | --- | --- |
| **Representation**  **and gene number**  **(CJB111 genome)** | **Gene name** | **Description** | **D1** | **D3** | **VG** | **CX** | **UT** |
| **Under-represented only in the uterus** | | | | | | | |
| ID870_01080 |  | Xaa-Pro dipeptidyl-peptidase |  |  |  |  | -3.592 |
| ID870_05485 | *pepF* | oligoendopeptidase F |  |  |  |  | -6.227 |
| ID870_06955 |  | aminodeoxychorismate/anthranilate synthase component II |  |  |  |  | -4.888 |
| ID870_05790 |  | MFS transporter |  |  |  |  | -6.678 |
| ID870_00140 |  | PTS transporter subunit IIBC |  |  |  |  | -4.922 |
| ID870_01090 |  | MFS transporter |  |  |  |  | -6.568 |
| ID870_03150 |  | hypothetical protein |  |  |  |  | -10.23 |
| ID870_05245 | *prsA* | peptidylprolyl isomerase |  |  |  |  | -6.465 |
| ID870_02760 | *gorA* | glutathione-disulfide reductase |  |  |  |  | -7.042 |
| ID870_02880 |  | NAD(P)/FAD-dependent oxidoreductase |  |  |  |  | -6.639 |
| ID870_02270 |  | aldo/keto reductase |  |  |  |  | -8.511 |
| ID870_08640 | *adcB* | metal ABC transporter permease |  |  |  |  | -6.371 |
| ID870_00465 |  | MFS transporter |  |  |  |  | -5.424 |
| ID870_07025 |  | magnesium transporter CorA family protein |  |  |  |  | -4.98 |
| ID870_08470 |  | ABC transporter permease (Opp/Pep transport) |  |  |  |  | -6.773 |
| ID870_08465 |  | ABC transporter ATP-binding protein (Opp/Pep transport) |  |  |  |  | -4.339 |
| ID870_04150 | *carA* | carbamoyl phosphate synthase small subunit |  |  |  |  | -9.824 |
| ID870_06630 |  | nucleoside hydrolase |  |  |  |  | -4.103 |
| ID870_03740 | *tpx* | thiol peroxidase |  |  |  |  | -15.55 |
| ID870_00575 |  | IS5 family transposase |  |  |  |  | -7.927 |
| ID870_02140 |  | class I SAM-dependent methyltransferase |  |  |  |  | -8.143 |
| ID870_10420 |  | HAMP domain-containing histidine kinase |  |  |  |  | -5.243 |
| ID870_01655 | *greA* | transcription elongation factor |  |  |  |  | -9.41 |
| ID870_09330 |  | MurR/RpiR family transcriptional regulator |  |  |  |  | -8.693 |
| ID870_10200 | *argR* | arginine repressor |  |  |  |  | -7.696 |
| ID870_03920 |  | RluA family pseudouridine synthase |  |  |  |  | -4.393 |
| ID870_01445 | *rsfS* | ribosome silencing factor |  |  |  |  | -6.551 |
| ID870_07425 |  | YlxQ-related RNA-binding protein |  |  |  |  | -6.962 |
| ID870_02635 |  | class I SAM-dependent methyltransferase |  |  |  |  | -22.06 |
| ID870_01555 |  | ECF-type riboflavin transporter substrate-binding protein |  |  |  |  | -3.897 |
| ID870_10025 |  | DUF4298 domain-containing protein |  |  |  |  | -21.62 |
| ID870_06145 |  | hypothetical protein |  |  |  |  | -10.55 |
| ID870_09940 |  | Bro-N domain-containing protein |  |  |  |  | -10.46 |
| ID870_02040 |  | DUF3397 domain-containing protein |  |  |  |  | -10.21 |
| ID870_10305 |  | Cof-type HAD-IIB family hydrolase |  |  |  |  | -9.015 |
| ID870_07335 |  | biotin transporter BioY |  |  |  |  | -8.848 |
| ID870_06490 |  | CsbD family protein |  |  |  |  | -7.743 |
| ID870_04080 |  | protein-ADP-ribose hydrolase |  |  |  |  | -6.385 |
| ID870_05665 |  | MBL fold metallo-hydrolase |  |  |  |  | -6.148 |
| ID870_02105 | *cvfB* | RNA-binding virulence regulatory protein |  |  |  |  | -4.297 |
| ID870_00035 |  | tRNA-Lys |  |  |  |  | -21.94 |
| ID870_06485 |  | hypothetical protein |  |  |  |  | -12.42 |
| ID870_09070 |  | tRNA-Thr |  |  |  |  | -12.17 |
| ID870_04035 |  | hypothetical protein |  |  |  |  | -9.366 |
| ID870_03760 |  | hypothetical protein |  |  |  |  | -6.07 |
| ID870_08095 |  | hypothetical protein |  |  |  |  | -12.2 |
| ID870_07090 |  | hypothetical protein |  |  |  |  | -7.621 |
