# Supplementary material for: Genomic Analyses Identify Manganese Homeostasis as a Driver of Group B Streptococcal Vaginal Colonization
Source: mBio. 2022 Jun 6;13(3):e00985-22. doi: 10.1128/mbio.00985-22 (PMC9239048; doi:10.1128/mbio.00985-22)
Supplement: TABLE S5 [file mbio.00985-22-s0010.docx]

| Primer name | 5’ 🡪 3’ Sequence |
| --- | --- |
| *mtsA* 5’ flank Forward | ATATCCCGGGTTATGTAGCAGATACATATATTTG |
| *mtsA* 5’ flank Reverse | ATGTATTCAACAAAATGCTCCTTTTAAAATTAATATAAG |
| *mtsA* 3’ flank Forward | AAAATTATAAGAGTGAGAATCGACATTG |
| *mtsA* 3’ flank Reverse | ATATTCTAGACTAGTCTCCTTGTCCTAAC |
| Spectinomycin Forward | GAGCATTTTGTTGAATACATACGAACAAATTAATAAAG |
| Spectinomycin Reverse | ATTCTCACTCTTATAATTTTTTTAATCTGTTATTTAAATAGTTTATAG |
| Spec chromosomal check Forward | GAATATTGAATGGACTAATGAAAATG |
| Spec chromosomal check Reverse | GTTTCCGAAGTATTTTTTTCA |
| 5’ *mtsA* chromosomal check Forward | GTATTAATGGCATTTTTGAAAG |
| 3’ *mtsA* chromosomal check Reverse | CAAAAAATGATAAGTCAATAAGC |
| *mtsA* complement 5’ Forward | ATATTCTAGAATGAAAAAGTGGTTAGTTATTG |
| *mtsA* complement 3’ Reverse | ATATGAATTCTTATTTTGCTAAACCTTCTGC |
| *gyrA* qPCR Forward | AGCACAAAAACGTGGAGGAC |
| *gyrA* qPCR Reverse | ACGATAGGGAGGCCTTTAGC |
| *mtsA* qPCR Forward | CATCGCTAACCGCAAAGTAATC |
| *mtsA* qPCR Reverse | AATCTTGAAACAGGCGGTAATG |
